# Investigation of effect of modulation frequency on high-density diffuse optical tomography image quality

**DOI:** 10.1117/1.NPh.8.4.045002

**Published:** 2021-11-24

**Authors:** Weihao Fan, Hamid Dehghani, Adam T. Eggebrecht

**Affiliations:** a Washington University, Department of Physics, St. Louis, Missouri, United States; b University of Birmingham, School of Computer Science, Birmingham, United Kingdom; c Washington University School of Medicine, Mallinckrodt Institute of Radiology, St. Louis, Missouri, United States; d Washington University, Department of Biomedical Engineering, St. Louis, Missouri, United States

**Keywords:** functional near-infrared spectroscopy, frequency domain high-density diffuse optical tomography, modulation frequency, point spread function, image quality, tissue optics, phase measurement, human brain imaging, simulation, system design, brain

## Abstract

**Significance:** By incorporating multiple overlapping functional near-infrared spectroscopy (fNIRS) measurements, high-density diffuse optical tomography (HD-DOT) images human brain function with fidelity comparable to functional magnetic resonance imaging (fMRI). Previous work has shown that frequency domain high-density diffuse optical tomography (FD-HD-DOT) may further improve image quality over more traditional continuous wave (CW) HD-DOT.

**Aim**: The effects of modulation frequency on image quality as obtainable with FD-HD-DOT is investigated through simulations with a realistic noise model of functional activations in human head models, arising from 11 source modulation frequencies between CW and 1000 MHz.

**Approach**: Simulations were performed using five representative head models with an HD regular grid of 158 light sources and 166 detectors and an empirically derived noise model. Functional reconstructions were quantitatively assessed with multiple image quality metrics including the localization error (LE), success rate, full width at half maximum, and full volume at half maximum (FVHM). All metrics were evaluated against CW-based models.

**Results**: Compared to CW, localization accuracy is improved by >40% throughout brain depths of 13 to 25 mm below the surface with 300 to 500 MHz modulation frequencies. Additionally, the reliable field of view in brain tissue is enlarged by 35% to 48% within an optimal frequency of 300 MHz after considering realistic noise, depending on the dynamic range of the system.

**Conclusions**: These results point to the tremendous opportunities in further development of high bandwidth FD-HD-DOT system hardware for applications in human brain mapping.

## 1 Introduction

Functional near-infrared spectroscopy (fNIRS) is widely used in functional brain mapping^1^ and neuro-monitoring at the bedside^2^^,^^3^ due to its portability, low expense, and noninvasiveness compared to traditional neuroimaging modalities such as functional magnetic resonance imaging (fMRI). The fNIRS technique is based on the differential absorption and scattering of NIR light associated with local changes in hemoglobin concentration.^4^ These two processes can be described by the absorption coefficient $\mu_{a}$ and the reduced scattering coefficient ${\mu_{s}}^{\prime }$. By acquiring multiple spatially overlapping fNIRS source—detector pairs, one can obtain image reconstructions of three-dimensional (3D) spatial brain activation maps, a technique known as diffuse optical tomography (DOT).^5^^–^^7^ Although early studies using sparse fNIRS systems demonstrated recovery of brain function with lower resolution and localization accuracy as compared to fMRI,^8^^–^^10^ recent advancements with high-density diffuse optical tomography (HD-DOT), which uses overlapping multi-distance source-detector pairs, have demonstrated significant improvements in the resolution and localization error (LE) of image reconstructions.^11^ More recent studies have shown potential further improvements in HD-DOT image quality and brain sensitivity when using high-bandwidth sinusoidal source modulation,^12^^,^^13^ a technique often referred to as frequency domain. However, the effects of modulation frequency on image quality with HD-DOT have yet to be elucidated.

Three dominant techniques of fNIRS are continuous wave (CW), frequency domain (FD), and time domain (TD) systems. The TD measurements use picosecond pulsed light sources and expensive high bandwidth single photon detectors. Arrival times of photons are converted into a distribution of time of flight (DTOF), which can then be used to derive both absorption and reduced scattering coefficients.^14^^–^^17^ With CW measurements, only the attenuation of NIR light from a continuous light source is used and therefore only differential changes in absorption can be recovered assuming a known and constant reduced scattering coefficient. In contrast, FD techniques use sinusoidally amplitude modulated light to measure changes in both intensity attenuation and phase delay relative to the source and therefore recover multiple differential contrasts (and, potentially, absolute measurements) of tissue optical properties including both the absorption coefficient $\mu_{a}$ and reduced scattering coefficient ${\mu_{s}}^{\prime }$. Due to the attenuation and phase measurements inherent to FD, these methods can provide superior image quality as compared to CW systems with an equivalent arrangement of sources and detectors.

A growing literature points to opportunities in leveraging the phase component of the FD measurements for improving image quality and brain sensitivity. Previous work investigating FD fNIRS has shown temporal phase measurements can recover concentration changes of hemoglobin by neglecting small scattering variations.^18^^,^^19^ Additionally, it has been shown that FD measurements can improve image quality and spatial resolution over CW measurements due to deeper sensitivity of measurements of phase delay in comparison to intensity attenuation alone.^12^ Moreover, this phase signal of FD measurements has been shown to be sensitive to brain signals at short source-detector separations that show no brain sensitivity in intensity attenuation measurements as used in CW.^13^ Further, more recent dual-slope methods have been shown to improve the phase sensitivity over single-slope methods in the case of localized inhomogeneous changes in optical properties.^20^^,^^21^ Additional techniques such as combining modulation frequencies may lead to better performance over CW,^22^ though care must be taken when removing superficial signals for FD measurements.^23^

Most extant studies on high density FD imaging performance have focused on one modulation frequency, typically around 100 to 200 MHz.^24^ However, some previous theoretical and experimental results point to tremendous opportunities to investigate high bandwidth (>200  MHz) source modulation frequencies. For example, one study using Monte Carlo simulations suggested that FD requires over 1 GHz modulation frequency to achieve significantly better spatial resolution than CW.^25^ However, in another study, it was shown in simulation and *in situ* that the highest instrument phase signal to noise is obtained in the range of 400 to 600 MHz.^26^ Given this literature, we aimed herein to investigate the potential improvements in FD-HD-DOT image quality over a wide range of modulation frequencies.

Here, we extend beyond previous studies to investigate the effects of modulation frequency on image quality over a range of modulation frequencies between CW (0 Hz) and 1 GHz. We simulated functional point activations at each voxel within the optically accessible tissue of five representative subject-specific head models with an HD grid of 158 sources and 166 detectors. To evaluate image quality within the context of current hardware specifications as well as potential future improvements, we simulated both systems with a moderate and strong dynamic range. Additionally, we simulated both noise-free cases and cases with an empirically derived noise model with respect to both source-detector distance and modulation frequency. We evaluated reconstructions with multiple quantitative metrics of resolution and localization accuracy throughout the 3D imaging domain. Additionally, we evaluated improvements in image quality between FD and CW as a function of depth and as a function of the volume of accessible brain tissue with successful image reconstructions. We hypothesized that image quality would improve with modulation frequency, that FD models would be more resilient to noise contamination than CW models, and that the amount of brain volume accessible would increase with modulation frequency.

## 2 Methods

### 2.1 HD-DOT Head Model

Informed consent was obtained and the research was approved by the Human Research Protection Office at Washington University School of Medicine. The head models were created as previously described.^1^ Briefly, the subject-specific models were based on five healthy young adult female participants with a mean age of 24 years (range 22–26 years). All participants passed MRI safety screening to ensure their safe participation. For each participant, we collected a T1- weighted MPRAGE (echo time = 3.13 ms, repetition time (TR) = 2400 ms, flip angle = 8 deg, $1\times 1\times 1\;{\mathrm{mm}}^{3}$ isotropic voxels) and a T2-weighted volume (TE = 84 ms, flip angle = 120 deg, 1×1×1  mm voxels). The T1-weighted MRI were segmented using FreeSurfer^27^^–^^29^ and combined with the T2-weighted volume for image segmentation in the NeuroDOT (https://github.com/WUSTL-ORL/NeuroDOT_Beta) software package into five tissue types: scalp (fat), skull (bone), cerebral spinal fluid (CSF), gray matter, and white matter, each with designated baseline optical properties (Table 1).^30^^–^^32^

**Table 1:** Optical properties used in 690- and 850-nm light modeling.

Tissues	$\mu_{a}$ (${\mathrm{mm}}^{-1}$)		$\mu_{s}^{\prime }$ (${\mathrm{mm}}^{-1}$)	
	690 nm	850 nm	690 nm	850 nm
Scalp^a^	0.0159	0.0190	0.8000	0.6400
Skull^a^	0.0101	0.0139	1.0000	0.8400
CSF^b^	0.0040	0.0040	0.3000	0.3000
Gray matter^c^	0.0201	0.0192	0.9727	0.6726
White matter^c^	0.0171	0.0208	1.3333	1.0107

a Strangman et al..

b Custo et al..

c Bevilacqua et al..

**Fig. 1:**
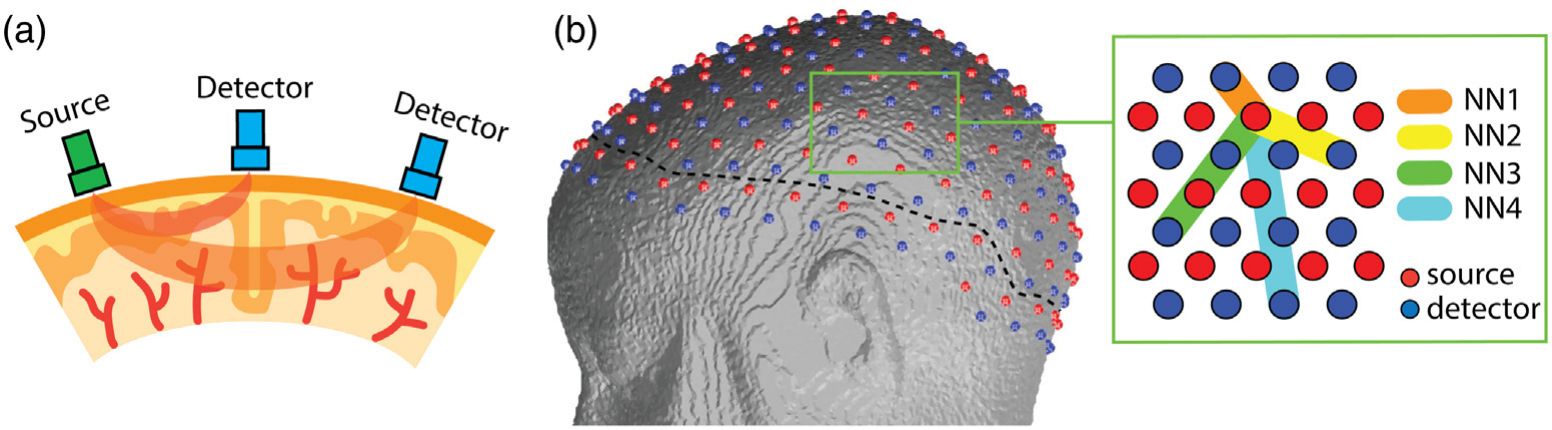
fNIRS measurements and example head model. (a) The distance between a light source and a detector effects the depth of sensitivity of the fNIRS measurement. (b) A representative head mesh based on an anatomical MRI with 158 sources (red) and 166 detectors (blue). The black dash line defines the ROI for PSF evaluation. The green box shows the first to fourth NN measurement topology with source-detector separations of approximately 13, 29, 39, and 47 mm, respectively.

High-resolution subject-specific finite element meshes, each consisting of five tissue layers were created, with mean element volume of $<1\;{\mathrm{mm}}^{3}$, resulting in ∼900,000 nodes and ∼5000,000 linear tetrahedral elements for each mesh using NIRFAST.^33^ The modeled HD-DOT pad consisted of 166 sources (red) and 158 detectors (blue) oriented on a representative head model as Figs. 1(a) and 1(b). The black dash line in Fig. 1(b) delineates the region of interest (ROI) to avoid edge effects of the grid array (1 cm inside the outer edge of optodes), beyond which the tomography is not well behaved.^34^ The green box illustrates the topography of the regular grid array of sources and detectors; measurements are labelled as NN1, NN2, NN3, and NN4, which respectively represent the first, second, third, and fourth nearest neighbors (NN) with average source-detector separation of 13, 29, 39, and 47 mm. For convenience, herein the labels of measurements involved in the simulations represent all the source-detector separations no larger than the labeling measurement. In other words, NN3 includes measurements of NN1, NN2, and NN3, whereas NN4 includes measurements of NN1, NN2, NN3, and NN4. The NN3 simulated system represents an HD-DOT system with moderate dynamic range and the NN4 represents one with strong dynamic range given the requirement of approximately an order of magnitude more dynamic range in light level detection required than the NN3 system.^1^^,^^7^

### 2.2 Forward Problem

In functional neuroimaging, our main goal is to find the relationship between the change in light measurements on the surface due to changes in tissue optical properties throughout the volume, which can be described as

y=A*x. (1)

Here, y represents differential measured data from source detector pairs, x refers to the differential changes of absorption coefficients and (possibly) reduced scattering coefficients of head tissues, and A is the sensitivity matrix or Jacobian that relates differential changes in optical properties within the volume to differential changes in the measurements on the surface. In the CW case, only intensity attenuation is considered in y, whereas for the FD case, y represents the variations of both light intensity and phase shift for each measurement.

**Fig. 2:**
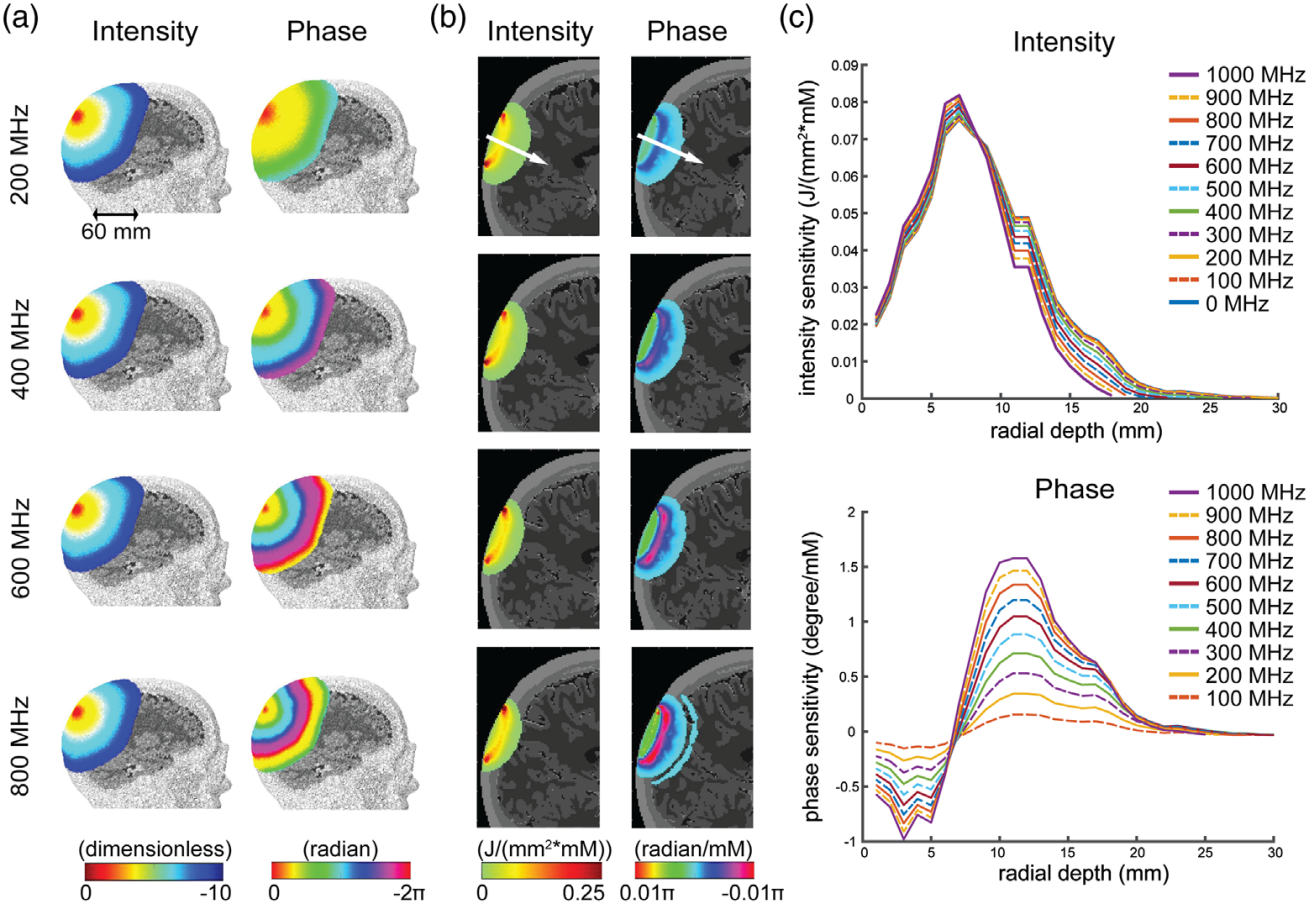
Effects of modulation frequency on FD measurement sensitivity in the five-layer model of the human head. (a) Normalized base-10 logarithm of the intensity attenuation (dimensionless) and phase (radians) distributions derived via Green’s functions from modeled 200, 400, 600, 800 MHz modulated light sources using baseline properties in Table 1. (b) Intensity sensitivity $\left[J/({\mathrm{mm}}^{2}\cdot \mathrm{mM}\right)$] and phase sensitivity (radians/mM) of a given source-detector measurement pair (39 mm separation) from a modeled 200, 400, 600, and 800 MHz modulated 850-nm light source. (c) The intensity (top) and phase (bottom) sensitivity changes as a function of depth as evaluated along the white arrow located at the midpoint of the source and detector in (b).

To obtain the relationship of Eq. (1) for this system, here we start with the diffusion equation (DE), a model based on the diffusive process of light propagation in tissue due to the high photon scattering in biological tissue,

$$(D(\overset{\to }{r})\nabla^{2}-v\mu_{a}(\overset{\to }{r})+i\omega )\Phi (\overset{\to }{r},\omega )=-vQ(\overset{\to }{r},\omega ),$$ (2)

where $\Phi (\vec{r},\omega )$ is photon fluence rate; ω=2πf is the angular frequency of the modulation; v is the speed of light in tissue; D represents the diffusion coefficient defined as $D=v/3(\mu_{a}+\mu_{s}^{\prime })$, where $\mu_{a}$ and ${\mu_{s}}^{\prime }$ are respectively the absorption and the reduced scattering coefficient where $\mu_{s}^{\prime }=(1-g)\mu_{s}$ with $\mu_{s}$ being the scattering coefficient. Here g=⟨cos θ⟩ is the anisotropic value, which is typically assumed as forward scattering having a value of 0.9 in biological tissue.^30^^,^^35^^,^^36^ The $\mu_{a}$ and ${\mu_{s}}^{\prime }$ coefficients represent the reciprocal of the mean distance traveled before a photon is absorbed/scattered in the absence of scattering/absorption. The source term,

$$Q(r,t)=Q_{DC}(r)+Q_{AC}(r)e^{-i\omega t},$$ (3)

describes the power per unit volume entering the system and includes components of direct current and altering current. We then transform the DE to a common form of the inhomogeneous Helmholtz equation as

$$(\nabla^{2}+\kappa^{2})\Phi (\overset{\to }{r},\omega )=\frac{v}{D}Q(\overset{\to }{r},\omega ),$$ (4)

where $\kappa =\sqrt{(-v\mu_{a}+i\omega )/D}$. We used NIRFASTer^37^ (https://github.com/nirfaster/NIRFASTer) to calculate Green’s function solutions to this equation given the morphology of the simulated human heads. Cross-sectional views of representative Green’s functions of a modulated point source on the back of a model of the human head [Fig. 2(a)] highlight that while modulation frequency moderately influences the light attenuation rate with distance from the source, the modulation frequency strongly influences the spatial pattern of the phase shift relative to the source position. In practice, a low modulation frequency (f<100  MHz) will cause nearly unmeasurably small phase shift measurements for source-detector separations <4  cm while a high modulation frequency (f>1  GHz) will induce a phase shift insensitive to optical properties.

Using the Rytov approximation, we can separate FD measurements into two parts with the complex logarithm where the real component represents the intensity attenuation and the imaginary component represents the phase shift at the detector,^38^

$$\delta \Phi =\ln(\frac{\Phi }{\Phi_{0}})=\ln(\frac{y_{I}e^{i\theta }}{y_{I0}e^{i\theta_{0}}})=\ln(\frac{y_{I}}{y_{I0}})+i(\theta -\theta_{0}),\;\;\;\;\;\;\;\;\;$$ (5)

where $\Phi_{0}$ is the baseline of modulated light, typically calculated as the temporal mean of the measurement. Combining this equation with Eq. (1), we can write the relationship between FD measurements with respect to changes of the absorption coefficient and the reduced scattering coefficient (or more colloquially, the diffusion coefficient) in matrix form as

[∂ ln yI∂yθ]=[Re(AΔμa)Re(AΔD)Im(AΔμa)Im(AΔD)][∂μa∂D]. (6)

Equation (6) indicates that the FD sensitivity matrix consists of real and imaginary components that convert optical property changes within the volume to intensity and phase measurements at surface respectively. The sensitivity matrix A depends on: (1) the geometry of head model; (2) the spatial distribution of optical properties of the head tissue; (3) and the distribution of sources and detectors along with their wavelength and modulation frequency. To calculate A, we used the NIRFASTer modeling software package to model Green’s functions of each source-detector measurement pair. Given that differential changes in scattering have yet to be well characterized in fNIRS or HD-DOT measurements of brain function, we will assume no scattering changes and thus we can further simplify Eq. (6) as

[∂ ln yI∂yθ]=[Re(AΔμa)Im(AΔμa)][∂μa]. (7)

Example sensitivity profiles for a modelled source-detector pair with 39 mm separation [Fig. 2(b)] further highlight that while the intensity profiles for different modulation frequencies are similar, the phase profiles highlight remarkably distinct and consistent patterns as the modulation frequency increases. Further examination along a depth profile perpendicular to the surface of the head at the midpoint of the measurement [along the white arrow in Fig 2(b), with values plotted in Fig 2(c)] reveals that the change in phase sensitivity with depth increases significantly as the modulation frequency increases, whereas the effect on the intensity profiles in depth are modest.

### 2.3 Inverse Problem

The process to reconstruct images is called the inverse problem because it converts relative changes in light intensity and phase into relative changes of absorption and diffusion at each wavelength. This step involves calculating the inverse of the Jacobian A in Eq. (6), which can be found approximately using a Moore–Penrose pseudoinverse with Tikhonov regularization:

A−1≈A#=L−1(A^TA^+λ1I)−1A^T, (8)

where $A^{\#}$ is Moore Penrose inverse of A; A^T denotes the transpose matrix of A^, and A^ is A spatially regularized by term L, which are defined as

A^=AL−1, (9)

$$diag(L)=\sqrt[{2}]{diag(A^{T}A)+\lambda_{2}}.$$ (10)

The parameters $\lambda_{1}$ and $\lambda_{2}$ are the Tikhonov regularization parameter and the spatially variant regularization parameter.^5^ The Tikhonov parameter $\lambda_{1}$ adjusts the balance between high spatial frequency noise and image smoothness and is set to 0.01 times the maximum singular value of A^TA^. The spatially variant regularization parameter $\lambda_{2}$ helps improve LE and is set to 0.1 times the maximum singular value of $A^{T}A$.^39^^,^^40^ To simplify, in this study, we are using these optimized regularization parameters of $\lambda_{1}$ and $\lambda_{2}$ as 0.01 and 0.1, respectively, based on previous studies, and invert the Jacobian separately for each wavelength.^39^^,^^41^

### 2.4 Realistic Noise Model

**Fig. 3:**
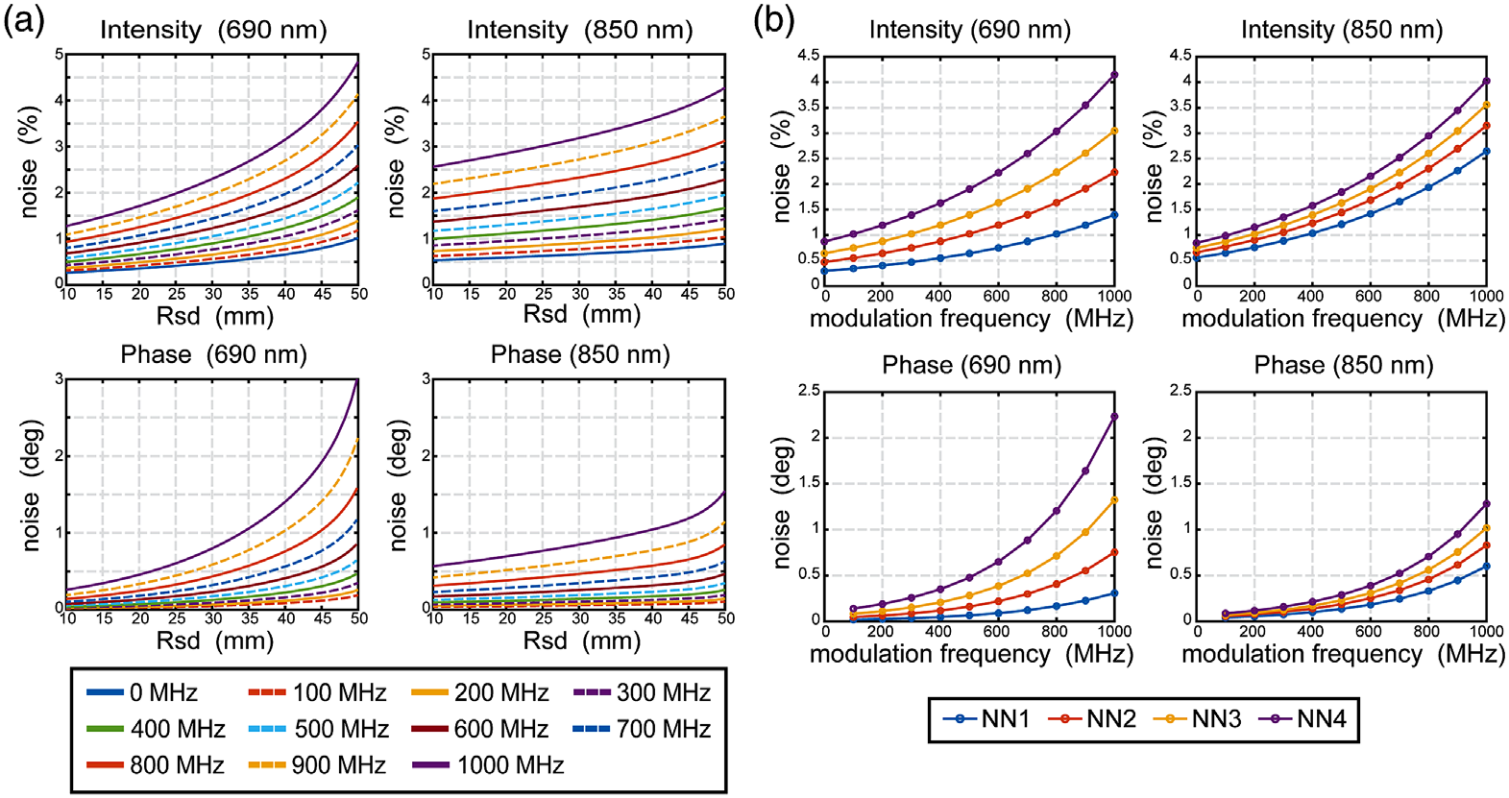
Noise model distributions. (a) Noise for each modulation frequency increases with increasing source-detector separation. (b) Noise for each source-detector separation (fits for 13, 29, 39, and 47 mm are shown) increases with increasing source modulation.

For general and broad interpretation of simulation studies, it is imperative to include a noise model in the simulations to evaluate if different modulation frequencies are robust to noise contamination in the measurements. It has been shown that noise in HD-DOT measurements are mainly due to (in decreasing order of magnitude) physiological variance and shot noise at high light level, and thermal detector noise at low light level.^26^ Previous work has shown that physiological variance in DOT data are caused by cardiac pulsation;^42^ respiration;^43^ low frequency oscillation of blood in vascular compartments of the brain and superficial tissues,^44^ each of which have characteristic frequency content; and motion induced noise signals that are generally broadband in nature.^45^^–^^47^ For experimental studies, it is common to use signal regression to remove nuisance signals due to systemic hemodynamic variance.^23^^,^^48^^,^^49^ Additionally, it is known that the noise level of FD measurements varies with the source-detector separation distance and is also affected by modulation frequency.^50^^,^^51^ Given that we are modeling source-detector measurements separated by up to 47 mm, the data are in the high light level regime and so we considered the physiological and shot noise based variance as dominating the thermal noise. Thus, we generated herein a noise model that combines separate empirical noise models as functions of source-detector separation and modulation frequency. First, a noise model as a function of source-detector distance was derived from data collected on the scalp during quiet rest.^12^ Second, we incorporated a recent noise model with respect to modulation frequency derived from phantom data.^51^ The full empirical realistic noise model N(r,f) is given by a two-term exponential function of source–detector distance r that is multiplied by the frequency-dependent noise model that is valid at an arbitrary modulation frequency f and normalized to a 140 MHz modulation frequency,

$$N(r,f)=(ae^{br}+ce^{dr})\cdot 10^{\left(g(f-140)\right)},$$ (11)

where the coefficients a, b, c, d, and g are derived from realistic data (Table 2; Fig. 3). The normalization is because the source-detector distance noise model was derived from data collected at 140 MHz.^12^ This combined noise model was added directly to the simulated measurements as

y=A*x+N. (12)

**Table 2:** Values of coefficients in realistic noise model.

Coefficients	Intensity noise model		Phase noise model	
	690 nm	850 nm	690 nm	850 nm
a^a^ (% or deg)	0.2502	0.6019	3.933e−11	1.917e-10
b^a^ (${\mathrm{mm}}^{-1}$)	0.02913	0.01052	0.4161	0.3708
c^a^ (% or deg)	4.625e−06	9.685e-05	0.0105	0.03573
d^a^ (${\mathrm{mm}}^{-1}$)	0.2128	0.1382	0.05585	0.02002
g^b^ (${\mathrm{MHz}}^{-1}$)	6.769e−04	6.785e−04	0.0013	0.0013

a Doulgerakis et al.

b Applegate et al.

The full noise model thus obeys a Gaussian distribution with zero mean and standard deviation sampled from N(r,f) (Fig. 3). The noise sources for intensity and phase for a given source-detector pair are coupled via matched random numbers for a given simulation.

### 2.5 Simulations

To quantitatively evaluate image quality, we modeled point spread functions (PSFs) arising from point perturbations within M≈190,000  voxels (per head model) within all tissues contained within optically accessible regions of the anatomical volume. The deep boundary of interrogation within the head was set to $10^{-5}$ of the maximum of the Green’s function fall-off in intensity for all sources and detectors to ensure a thorough sampling in the volume (corresponding to approximately 35.3 mm below the surface). We also constrained our investigation to the ROI contained within 1 cm or more from inside the edge of the HD-DOT array to avoid edge effects [dotted black line in Fig. 1(b)]. Throughout this ROI, we simulated point perturbations ∂Hb of 3.8   μM for oxygenated hemoglobin (${\mathrm{HbO}}_{2}$) and −1.8  μM for deoxygenated hemoglobin (HbR), values that are consistent with previous simulations based on similar FD HD-DOT designs.^12^ These perturbations in ${\mathrm{HbO}}_{2}$ and HbR were spectroscopically converted into perturbations in absorption $\partial \mu_{a}$ at source illumination wavelengths of λ=690  nm and λ=850  nm via,^52^^,^^53^

$$[\begin{array}{c} \partial \mu_{a,690}\\ \partial \mu_{a,850} \end{array}]=[\begin{array}{cc} \varepsilon_{HbO_{2},690} & \varepsilon_{HbR,690}\\ \varepsilon_{HbO_{2},850} & \varepsilon_{HbR,850} \end{array}][\begin{array}{c} \partial HbO_{2}\\ \partial HbR \end{array}],$$ (13)

using tabulated molar extinction coefficients,^54^ where, $\varepsilon_{c,\lambda }$ represents extinction coefficient of c (${\mathrm{HbO}}_{2}/\mathrm{HbR}$) for wavelength λ. These wavelengths have been previously shown to be maximally sensitive to activations in HbR and ${\mathrm{HbO}}_{2}$. These wavelength dependent changes in absorption ($\partial \mu_{a}=x_{\text{sim}}$) thus provided our simulated measurements via Eq. (12), $y_{\text{sim}}=Ax_{\text{sim}}+N$. The reconstructed volumetric wavelength-dependent perturbation in absorption is thus given by $x_{\mathrm{recon}}=A^{\#}y_{\text{sim}}=A^{\#}(Ax_{\text{sim}}+N)$, which is then propagated through the inverse of Eq. (13) to arrive at the PSF for ${\mathrm{HbO}}_{2}$ and HbR. To simplify, we focus our analyses on PSFs for ${\mathrm{HbO}}_{2}$, though results for HbR are qualitatively similar and are presented in Figs. S1–S6 in the Supplementary Materials. These simulations were run in ∼190,000  voxels per head model, over five head models, for frequencies including: 0, 100, 200, 300, 400, 500, 600, 700, 800, 900, and 1000 MHz.

### 2.6 Analyses

To quantitatively evaluate the image quality of the simulated reconstructions, we threshold the recovered PSF at half maximum and then calculated the full width at half maximum (FWHM), the full volume at half maximum (FVHM), LE, and success rate (SR). The FWHM is the longest distance between any two voxels included in PSF. The FVHM is the total volume of the PSF. This is a complementary metric to the FWHM as the cube root of the FVHM can be somewhat better behaved as a metric of the linear size of the PSF. The LE is calculated as the distance between the perturbation point and centroid of the PSF (Tables S1 and S2 in the Supplementary Materials). The SR is defined as the percentage of voxels at a given depth with LE less than the 8-mm cutoff. We only consider FWHMs and FVHMs for voxels whose LEs are at most 8 mm. The depth at which a 50% SR is observed is thus defined as the depth boundary for reliable imaging.^12^^,^^34^ To study the improvement in spatial sensitivity to brain tissue using FD as compared to CW, we defined a new metric called the full brain tissue volume (FBTV) given as the total volume of brain tissue with a LE lower than 8 mm. Finally, we calculated the full brain tissue ratio (FBT ratio), given by the ratio of brain volume successfully recovered by FD (i.e., the FBTV) to that recovered by CW. These metrics quantify the image quality in aspects of spatial resolution, localization accuracy, and the extent of the reliable field of view both in absolute terms and relative to performance by the simulated CW system. To evaluate these metrics statistically between each simulated FD case and CW, we used the Wilcoxon signed rank test (Tables S3 and S4 in the Supplementary Materials). For example visualizations, we spatially normalized the five head models using a 3D affine transformation and averaged the metrics for a given voxel across the aligned head models.

## 3 Results

**Fig. 4:**
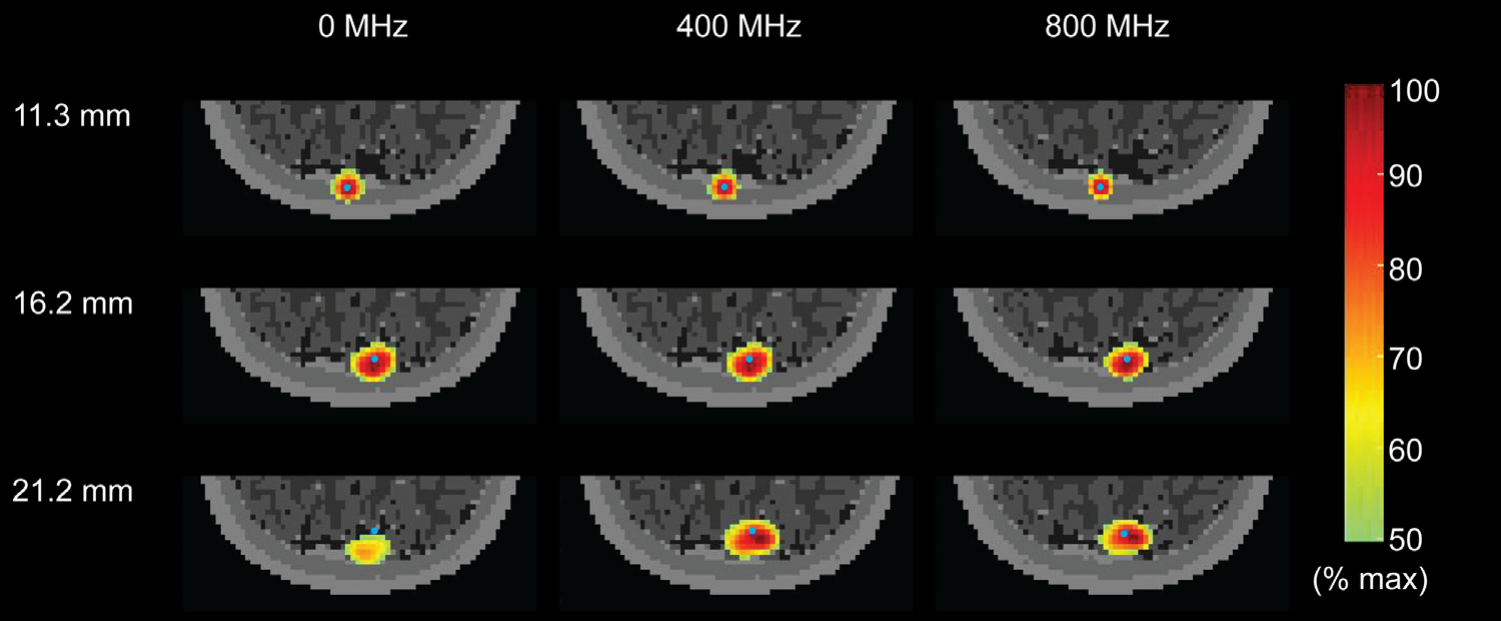
Examples of noise-free reconstructed PSFs. The PSFs are induced by simulated point activations at three depths, using NN3 measurements at 0, 400, and 800 MHz without noise. PSFs are normalized and cut off at 50% maximum. The blue dots denote the locations of point perturbations. Image metrics for these example PSFs are reported in Table 3.

**Fig. 5:**
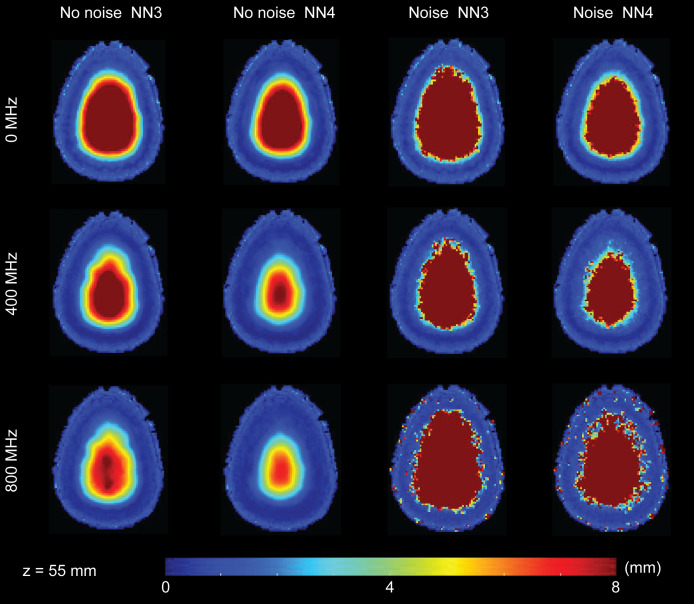
LE distribution in an example transverse slice. The color of each voxel reflects the LE in mm averaged across five head models after spatial alignment. For brevity, three modulation frequencies are shown: 0, 400, and 800 MHz.

**Fig. 6:**
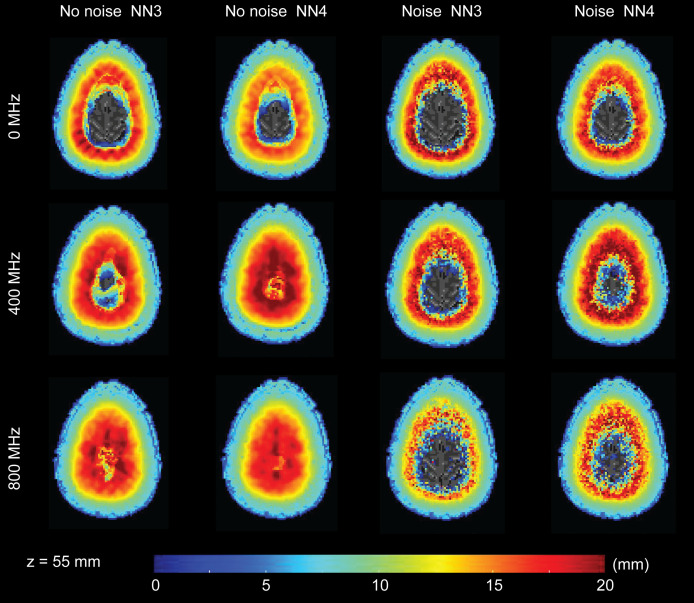
FWHM distribution in an example transverse slice. The color of each voxel reflects the FWHM in mm averaged across five head models after spatial alignment. For brevity, three modulation frequencies are shown: 0, 400, and 800 MHz.

**Fig. 7:**
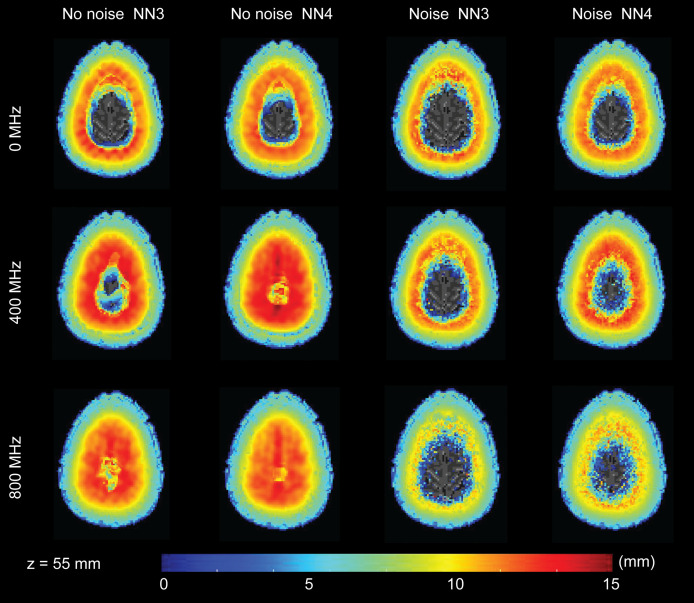
Spatial distribution of the cube root of the FVHM in an example transverse slice. The color of each voxel reflects the cube root of the FVHM in mm averaged across five head models after spatial alignment. For brevity, three modulation frequencies are shown: 0, 400, and 800 MHz.

To provide an overview of the spatial distribution characteristics of the recovered PSFs, consider three points in occipital cortex (Fig. 4). These focal simulated activations (blue dots) are modeled at depths of about 11, 16, and 21 mm. The reconstructed PSFs are shown for the noise-free CW case (0 MHz), 400 MHz and 800 MHz. The PSFs were filtered by half maximum value of the recoveries and normalized to their maximum value. Volumetric views of PSFs reveal two primary characteristics: (1) while the LE of the PSF increases with depth, the LE decreases with increasing modulation frequency (Table 3); (2) while the size of the PSF increases in depth, again as expected, the size of the PSF is only modestly affected by the modulation frequency (Table 3). For each modulation frequency, to quantitatively evaluate image quality throughout the optically accessible tissue in five head models, we reconstructed PSFs induced at each voxel throughout the ROI. We then calculated the LE at every voxel throughout the ROI. At each voxel with a LE <8  mm, considered a successful recovery, we calculated the resolution through the FWHMs and FVHMs. Representative slices through the volume show the spatial distributions of the mean LE (Fig. 5), FWHM (Fig. 6), and cube root of the FVHM (Fig. 7), with metrics averaged across the five head models after spatial alignment.

**Table 3:** Image metrics of the example noise free PSFs in Fig. 4.

Depth (mm)	0 MHz			400 MHz			800 MHz		
	LE	FWHM	FVHM	LE	FWHM	FVHM	LE	FWHM	FVHM
11.3	0.35	13.27	10.72	0.31	12.81	10.00	0.30	12.00	9.22
16.2	3.11	18.22	13.39	1.30	19.08	14.37	0.87	17.20	13.24
21.2	7.67	20.49	14.08	2.94	23.58	16.03	2.08	21.26	14.70

All metrics are in units of mm. Localization error (LE). Full width at half maximum (FWHM), cube root of full volume at half maximum (FVHM).

The LE data in Fig. 5 highlight a number of characteristics of the results. First, as expected in optical imaging, the LE increases with depth for all cases. Second, increasing the number of available measurements in the reconstruction (i.e., using NN4 instead of NN3) improves the LE. Third, the presence of noise adversely effects the LE. These first three observations are consistent regardless of modulation frequency. Fourth, while increasing the modulation frequency improves the LE for the noise free case, the effect of noise is stronger at the higher modulation frequency and leads to worse performance in LE. Indeed, the LE for the moderate FD case (400 MHz) appears to be less influenced by the presence of noise than for the CW case or the high frequency case (800 MHz).

The transverse slices of FWHM (Fig. 6) and cube root of FVHM (Fig. 7) allow us to visualize some of the effects of modulation frequency on spatial resolution. As with the LE in Fig. 5, we observe some characteristics of these resolution measures that are consistent across modulation frequency: resolution gets worse in depth, having more available channels improves resolution, and noise negatively effects resolution. Additionally, while there is some general improvement in resolution with frequency, the improvement is far more modest than that seen with LE.

**Fig. 8:**
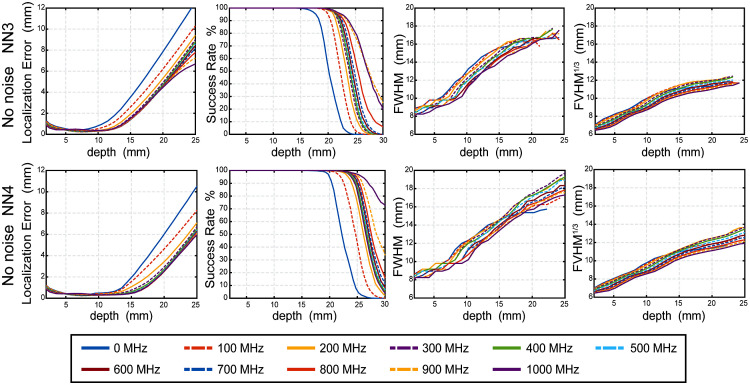
Image quality metrics as a function of depth below the surface in noise free cases. The median of the LE, success rate FWHM, cube root of FVHM across five head models’ simulated measurements of 11 modulation frequencies without noise added for NN3 and NN4. Even frequencies are solid lines and odd frequencies are dashed lines.

**Fig. 9:**
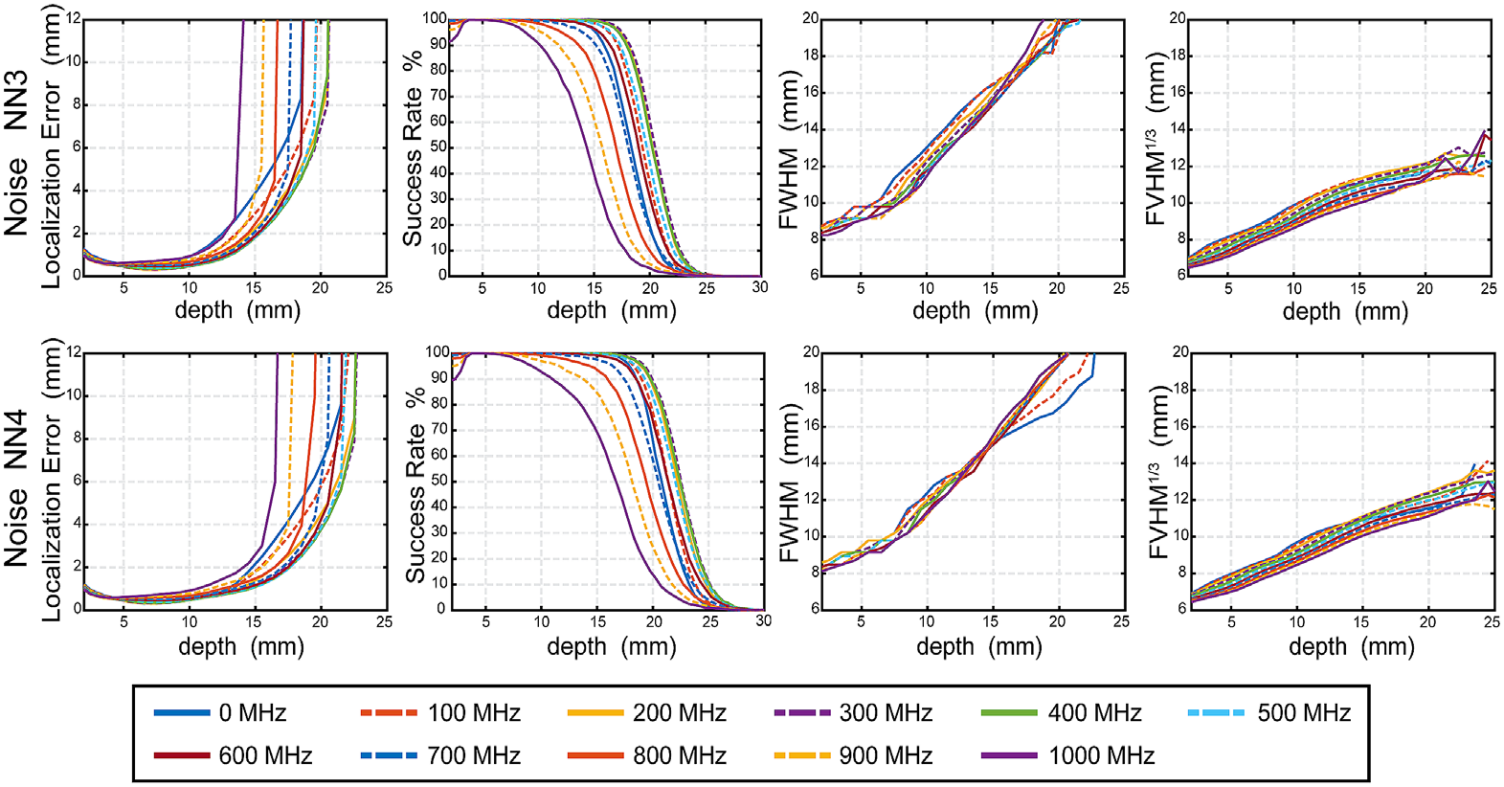
Image quality metrics as a function of depth below the surface in noise added cases. The median of the LE, success rate FWHM, cube root of FVHM across five head models’ simulated measurements of 11 modulation frequencies with noise added for NN3 and NN4. Even frequencies are solid lines and odd frequencies are dashed lines.

**Fig. 10:**
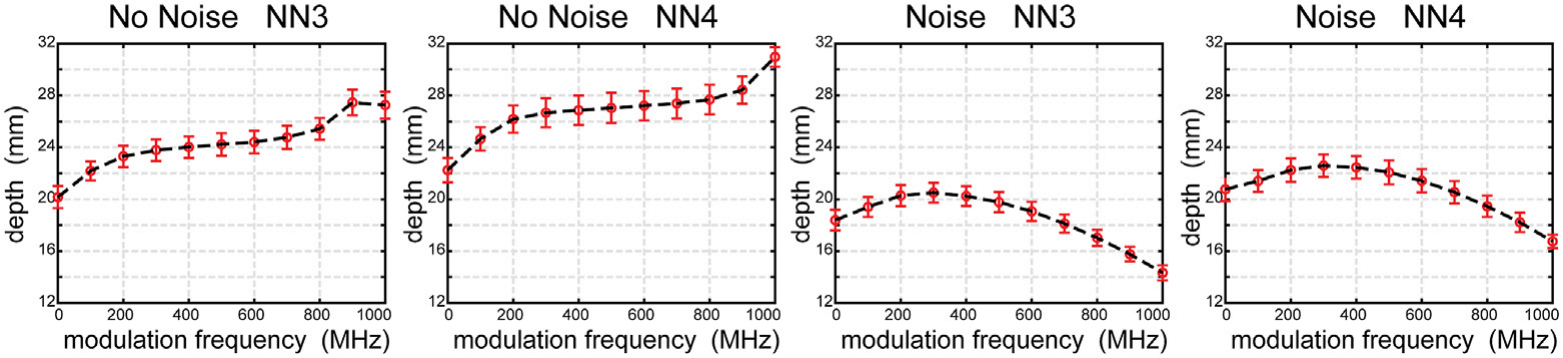
Depth of the reliable field of view across all modulation frequencies and noise models. Averaged depths of the 50% success rate as a function of modulation frequency. Error bars represent standard deviations across five head models.

To investigate the effect of tissue depth on the metrics, we present here results of LE, FWHM, and cube root of FVHM as lowest curve medians from data integrated over the five head models for the noise free case (Fig. 8) and the noise added case (Fig. 9). To define the boundary of the reliable imaging volume, we calculated success rate as a function of depth (second from left columns of Figs. 8 and 9) and extracted mean depth with 50% success rate over 5 subjects (Fig. 10). For greater detail, the medians, along with 25% and 75% values, for each metric for each frequency are presented binned by depth in S1-S4 in the Supplementary Materials.

The behaviors of LE, SR, FWHM, and cube root of FVHM as a function of depth in the 11 frequencies without noise are shown in Fig. 8. Generally, the FD modulation improves LE significantly, with improvement over CW increasing with modulation frequency. For example, at 13–18 mm depth, 400 and 800 MHz, respectively, provide improvements over CW mode by 55.45% and 56.98% in NN3 measurements, by 66.92% and 75.49% in NN4 measurements. However, the improvement in resolution with higher modulation frequency is modest. For example, 400 and 800 MHz respectively improve FWHM over CW mode by 2.57% and 7.02% in NN3 measurements; by 0% and 4.84% in NN4 measurements. The results are summarized in Tables S1 and S3 in the Supplementary Materials. The maximum improvement of LE is given by 800 MHz for NN3 and 1000 MHz for NN4, and maximum improvement of FWHM and FVHM are both given by 1000 MHz for NN3 and NN4. The results are more nuanced when considering the addition of the noise model in simulation (Fig. 9): We find the presence of noise causes the LE to increase rapidly around 15 to 23 mm due to more noise, especially at high modulation frequency above 500 MHz. As a result, optimum modulation frequencies for localization error are 400 MHz for NN3 and 300 to 400 MHz for NN4. Additionally, the FWHM and FVHM with noise are larger than counterparts without noise as expected, with the improvements at higher modulation frequency strongly diminished. Additionally, the SR drops to more shallow depths in all cases. The full results are summarized in Tables S2 and S4 in the Supplementary Materials.

To evaluate SR for reconstructions across the modulation frequencies, the mean (with standard deviation) depth of the 50% success rate across the five head models is shown for noise free and noise added cases (Fig. 10). As expected and observed above, the presence of noise lowers the successful imaging depth in all cases. Interestingly, the optimized frequency differs between noise free and noise added cases. In the noise free case, the deepest 50% SR boundary is 27.46±0.98  mm for NN3 given by 900 MHz and 30.97±0.75  mm for NN4 given by 1000 MHz. With the realistic empirical noise model, the deepest 50% success rate boundary is 20.50±0.76  mm for NN3 and 22.59±0.87  mm for NN4 both given by 300 MHz.

**Fig. 11:**
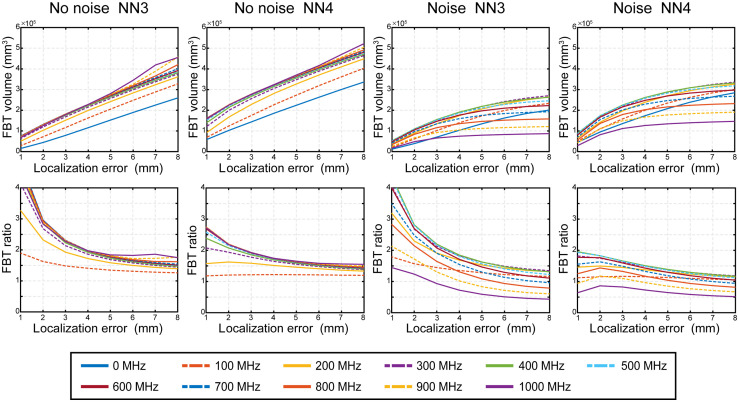
Improvement of reliable field of view in brain tissue by FD across LE thresholds. Brain volume (top) and brain volume relative to CW (bottom) averaged across five subjects at 11 modulation frequencies when LE limitation is set from 1 to 8 mm. Solid lines represent even frequencies and dash lines represent odd frequencies.

To quantify the improvement in image quality specifically within brain tissue of FD over CW, we calculated the full FBTV with successful reconstructions and the ratio of FBTV recoverable with a given modulation frequency to that recoverable with CW (FBT ratio). In Fig. 11 we show how the FBTV and FBT ratio behave with LEs ranging from 1 to 8 mm. From the figure, we observe that FBTV increases approximately linearly in the noise free cases but increases more slowly in the noise added cases. In the range of LE from 6 to 8 mm, we found that higher modulation frequency provides larger FBTV and FBT ratios in noise free cases where 900-MHz mode provides about 1.76 times and 1000 MHz mode provides 1.55 times larger FBT volume than CW mode respectively for NN3 and NN4. After adding noise, the optimum frequency mode is 300 MHz for both NN3 and NN4, which respectively provides about 1.35 times and 1.18 times larger volumetric access to brain tissue than the CW mode.

## 4 Discussion

The availability of CW-HD-DOT systems is increasing rapidly for both adult and pediatric studies owing to the demonstrated improvements in image quality obtained with HD arrays.^1^^,^^12^^,^^13^^,^^55^^–^^62^ Most research groups still utilize CW systems for wide-field optical imaging of human brain function as these systems are significantly less expensive than FD and TD systems. However, recent studies have highlighted a renewed focused on HD arrangements of FD measurements due to the value in incorporating the additional phase measurements to realize the potential for improved image quality and potential access to absolute oxygenation concentration.^12^^,^^13^^,^^20^^,^^22^ In addition, some studies theoretically demonstrate that an FD system can provide enhanced depth sensitivity using phase information. Herein, we extended previous work with simulations of HD-FD-DOT measurements with frequencies of 0, 100, 200, 300, 400, 500, 600, 700, 800, 900, and 1000 MHz and quantitatively evaluated image quality for each modulation frequency. The goal of our work was to investigate the potential of higher source modulation frequency in an HD arrangement and to find the optimum frequency range to guide future design of FD-HD-DOT instruments given the potential for improvements in image quality and brain sensitivity and specificity.

We hypothesized that higher modulation frequencies would provide better spatial resolution and LE as well as access to more brain volume than CW. The noise-free simulation results support our hypothesis on resolution and LE improvement, though resolution is improved only modestly. We can understand the improvement in spatial specificity through the observation that the spatial sensitivity profile in the phase measurement [Figs. 2(b) and 2(c)] exhibits a steeper and stronger magnitude change with increasing modulation frequency. This leads to greater spatial specificity (i.e., a lower LE) after tomographically combining the multi-distance overlapping measurements. Thus, while the location of the centroid of the PSF is more accurate with FD than CW, the width of the PSF (the resolution) is only modestly improved. To extend the analyses to more real-world situations, we added an empirical noise model that reflected both noise as a function of source-detector separation^12^ and as a function of modulation frequency^51^ (Fig. 3). The simulations incorporating this noise model clearly show the deleterious effects of system noise at high modulation frequencies that swamp out the added benefits of the narrowing and strengthening of the phase sensitivity. Nevertheless, these competing processes point to an optimum range of modulation frequencies around 300 MHz for image quality and depth sensitivity for an FD-HD-DOT system.

To highlight the spatial profile of image quality throughout the ROI, we showed transverse distributions of the LE, FWHM, and cube root of FVHM for example frequencies of 0, 400, and 800 MHz (Figs. 5–7). We also set the successful LE limit to 8 mm to define a reliable imaging region and provide a benchmark for meaningful statistics. In summary, the modulation frequency contributes most to improvement in localization accuracy but has less of an effect on spatial resolution, in agreement with the implications Fishkin and Gratton’s work.^25^ To highlight significant advantages in system designs with a larger dynamic range, we simulated both NN3 and NN4 measurement sets. In general, for all our simulations, the results are consistent with previous work about FD-HD-DOT system utilizing 140-MHz frequency in comparison to CW.^12^ The larger source-detector separations (especially >4  cm) provide access to deeper penetrating photons that sample larger volumes of brain tissue. However, obtaining strong signal to noise at these longer distances while simultaneously recording signals from shorter source separations places challenging demands on dynamic range that directly effects detector design, source encoding strategies, and crosstalk specifications.

To estimate the resultant reliable imaging region, we calculated the mean depth of 50% success rate over each head as well as the total brain volume with good image metrics. In the noise free case, 700 MHz gives the best LE and 1000 MHz gives best FWHM and FVHM for NN3; and 1000 MHz performs best for all metrics. In the noise added case, 300 MHz performs best for NN3 and for NN4. In summary, with the consideration of localization accuracy, spatial resolution, and reliable imaging volume, the optimum modulation frequency is in range of 300 MHz for an FD-HD-DOT system. Indeed, at higher modulation frequencies, the effects of phase wrapping may be unavoidable and provide a maximal depth penetration for a given FD frequency. In summary, a modulation frequency around 300 MHz may be an optimal target for future hardware development.

There are some limitations to note in this study. First, due to the huge computational cost, our study includes only five head models with eleven modulation frequencies. Future studies may extend these methods to cases with different anatomical morphology in adults, such as increased CSF due to cortical atrophy associated with aging or to younger children for whom deeper penetration of sensitivity will lead to much improved access to brain volume. Additionally, in general, the development of system design for FD systems is complex and ongoing with various options that each lead to drastically different noise spectra across modulation frequencies.^51^^,^^63^ While a full discussion of the hardware challenges with possible solutions is beyond the scope of the present work, we show herein the potential for FD imaging given further developments in FD system design that can lower the noise in high bandwidth systems. In addition, more elaborate models of the physiological noise component, as could be explained using the local covariance of each source-detector pair measurement, may further improve management of noise and therefore improve image quality. The second limitation is that optimization for a given modulation frequency may depend of different choices of regularization parameters^64^^–^^66^ or conditioning procedures for the Jacobian.^55^^,^^67^^–^^71^ A full exploration of these computational strategies and their effects at different modulation frequencies is beyond the scope of this paper. Last, here we were focused on optimizing for image quality of differential measurements of brain function. The results presented may not necessarily generalize to recovery of estimation of baseline optical properties.^51^ Future studies may explore high-bandwidth source modulation in high density measurement arrangements for such purposes. In general, our work demonstrates the significance of the potential for higher modulation frequency strategies than typically used in existing FD systems for future design of FD-HD-DOT systems.

In summary, we set out to investigate potential improvements in image quality in HD-DOT methods focusing on an optimal modulation frequency strategy. For simplicity, in this study, we focused on a range of single modulation frequencies. However, previous studies point to compelling opportunities afforded by combining multiple modulation frequencies in FD-HD-DOT system to improve the image quality,^22^^,^^71^ including the many potential methods for pre-conditioning the sensitivity matrix for multi-frequency DOT in addition to the careful selection of frequency combinations.^71^ Indeed, the promise of these techniques has been realized in hardware with a multi-frequency DOT instrument^72^ with applications in cancer detection.^73^ Potentially such methods will also translate to superior image quality, improved depth sensitivity, and functional specificity in brain imaging.

## 5 Conclusions

We report a systematic evaluation of image quality as a function of modulation frequency in FD-HD-DOT systems. Volumetric views of LEs and resolution throughout the full head volume provide a window into how source modulation frequency influences imaging fidelity and that higher frequency provides better localization accuracy and access to deeper regions with reliable imaging. In this quantitative study, we evaluated image quality of FD-HD-DOT in aspects of localization accuracy, spatial resolution, and volume of reliable field of view. As a result, we found that in noise-free models, image quality improves with increasing modulation frequency up to 1 GHz, whereas in realistic noise-added models, 300 MHz provides a target modulation frequency to optimize image quality metrics. This work provides specific motivations and targets for future development of FD hardware for HD-DOT systems for mapping human brain function.
