# Supplementary material for: Investigation of effect of modulation frequency on high-density diffuse optical tomography image quality
Source: Neurophotonics. 2021 Nov 24;8(4):045002. doi: 10.1117/1.NPh.8.4.045002 (PMC8612746; doi:10.1117/1.NPh.8.4.045002)
Supplement: Supplementary file 1 [file NPh_008_045002_SD001.pdf]

Supplementary materials for

Investigation of Effect of Modulation Frequency on High-Density Diffuse Optical Tomography Image Quality

Weihao Fan[1], Hamid Dehghani[2], Adam T. Eggebrecht[3,4]

[1]*Department of Physics, Washington University, St. Louis, MO, 63130, USA*

[2]*School of Computer Science, University of Birmingham, UK*

[3]*Mallinckrodt Institute of Radiology, Washington University School of Medicine, St. Louis, MO, 63130, USA*

[4]*Department of Biomedical Engineering, Washington University, St. Louis, MO, 63130, USA*

Contents

Supplementary Tables

Supplementary Table S1: Quantitative metrics in noise free models **2**
Supplementary Table S2: Quantitative metrics in noise added models **3**
Supplementary Table S3: Quantitative comparison of noise free FD models to CW **4**
Supplementary Table S4: Quantitative comparison of noise added FD models to CW **5**

Supplementary Figures

Supplementary Figure S1: Volumetric view of localization error distribution slices of HbR **6**
Supplementary Figure S2: Volumetric view of FWHM distribution slices of HbR **7**
Supplementary Figure S3: Volumetric view of cube root of FVHM distribution slices of HbR **8**
Supplementary Figure S4: Quantitative metrics vs. depth in noise free models for HbR **9**
Supplementary Figure S5: Quantitative metrics vs. depth in noise added models for HbR **9**
Supplementary Figure S6: Depth of 50% success rate as function of modulation frequency for HbR **10**

Supplementary Table S1 Medians, 25th (subscript) and 75th (superscript) percentiles of localization error, FWHM, FVHM (mm) of all FD modes to CW mode at different depth intervals without noise model.

Depth (mm)	Frequency(MHz)	Localization error		FWHM		FVHM	
		NN3	NN4	NN3	NN4	NN3	NN4
3 to 8	0	$0.53_{0.30}^{0.79}$	$0.40_{0.23}^{0.68}$	$9.80_{9.80}^{10.77}$	$9.80_{9.17}^{9.80}$	$8.28_{7.83}^{8.65}$	$8.08_{7.65}^{8.51}$
	100	$0.44_{0.25}^{0.68}$	$0.39_{0.22}^{0.66}$	$9.80_{9.17}^{10.20}$	$9.80_{9.17}^{9.80}$	$8.20_{7.74}^{8.55}$	$8.04_{7.61}^{8.47}$
	200	$0.38_{0.22}^{0.62}$	$0.37_{0.22}^{0.62}$	$9.80_{9.17}^{9.80}$	$9.38_{9.17}^{9.80}$	$8.04_{7.56}^{8.51}$	$7.92_{7.47}^{8.43}$
	300	$0.36_{0.22}^{0.57}$	$0.36_{0.22}^{0.57}$	$9.38_{9.17}^{9.80}$	$9.17_{8.94}^{9.80}$	$7.87_{7.42}^{8.40}$	$7.79_{7.37}^{8.32}$
	400	$0.37_{0.24}^{0.54}$	$0.36_{0.23}^{0.54}$	$9.17_{8.94}^{9.80}$	$9.17_{8.94}^{9.80}$	$7.74_{7.27}^{8.28}$	$7.70_{7.27}^{8.24}$
	500	$0.39_{0.26}^{0.54}$	$0.37_{0.24}^{0.53}$	$9.17_{8.94}^{9.80}$	$9.17_{8.94}^{9.80}$	$7.61_{7.17}^{8.20}$	$7.61_{7.11}^{8.08}$
	600	$0.40_{0.27}^{0.55}$	$0.39_{0.25}^{0.54}$	$9.17_{8.94}^{9.80}$	$8.94_{8.72}^{9.38}$	$7.51_{7.01}^{8.08}$	$7.47_{6.95}^{7.96}$
	700	$0.42_{0.28}^{0.57}$	$0.40_{0.27}^{0.55}$	$8.94_{8.94}^{9.38}$	$8.94_{8.49}^{9.17}$	$7.42_{6.90}^{7.96}$	$7.37_{6.84}^{7.87}$
	800	$0.44_{0.30}^{0.58}$	$0.42_{0.28}^{0.56}$	$8.94_{8.94}^{9.17}$	$8.94_{8.25}^{9.17}$	$7.32_{6.78}^{7.87}$	$7.22_{6.72}^{7.74}$
	900	$0.45_{0.30}^{0.59}$	$0.43_{0.28}^{0.56}$	$8.94_{8.25}^{9.17}$	$8.94_{8.25}^{9.17}$	$7.22_{6.72}^{7.79}$	$7.11_{6.66}^{7.65}$
	1000	$0.46_{0.30}^{0.60}$	$0.43_{0.28}^{0.57}$	$8.94_{8.25}^{9.17}$	$8.94_{8.25}^{9.17}$	$7.11_{6.66}^{7.70}$	$7.01_{6.60}^{7.51}$
8 to 13	0	$0.92_{0.52}^{1.48}$	$0.51_{0.28}^{0.78}$	$13.12_{12.00}^{14.14}$	$12.33_{11.49}^{13.27}$	$10.05_{9.41}^{10.63}$	$9.84_{9.13}^{10.46}$
	100	$062_{0.35}^{0.97}$	$0.46_{0.25}^{0.69}$	$12.81_{12.00}^{14.00}$	$12.17_{11.49}^{13.27}$	$10.05_{9.25}^{10.67}$	$9.73_{9.06}^{10.39}$
	200	$0.41_{0.23}^{0.66}$	$0.38_{0.21}^{0.57}$	$12.65_{11.49}^{13.57}$	$12.17_{11.31}^{13.12}$	$9.84_{9.06}^{10.58}$	$9.55_{8.93}^{10.26}$
	300	$0.36_{0.20}^{0.56}$	$0.33_{0.19}^{0.49}$	$12.17_{11.31}^{13.27}$	$12.00_{10.77}^{12.81}$	$9.58_{8.90}^{10.39}$	$9.34_{8.83}^{10.05}$
	400	$0.34_{0.21}^{0.53}$	$0.30_{0.18}^{0.44}$	$12.00_{10.77}^{13.27}$	$11.66_{10.20}^{12.65}$	$9.34_{8.79}^{10.18}$	$9.16_{8.72}^{9.86}$
	500	$0.34_{0.22}^{0.51}$	$0.29_{0.18}^{0.42}$	$12.00_{10.20}^{12.81}$	$11.49_{10.20}^{13.33}$	$9.19_{8.69}^{9.97}$	$9.00_{8.62}^{9.64}$
	600	$0.35_{0.23}^{0.51}$	$0.29_{0.19}^{0.42}$	$11.49_{10.20}^{12.81}$	$11.31_{9.80}^{12.17}$	$9.06_{8.58}^{9.81}$	$8.86_{8.55}^{9.47}$
	700	$0.36_{0.24}^{0.52}$	$0.30_{0.20}^{0.42}$	$11.49_{10.20}^{12.65}$	$10.77_{9.80}^{12.00}$	$8.93_{8.55}^{9.67}$	$9.76_{8.47}^{9.31}$
	800	$0.37_{0.25}^{0.54}$	$0.30_{0.21}^{0.43}$	$11.31_{9.80}^{12.33}$	$10.20_{9.80}^{12.00}$	$8.86_{8.47}^{9.55}$	$8.69_{8.40}^{9.19}$
	900	$0.39_{0.26}^{0.57}$	$0.32_{0.21}^{0.45}$	$11.31_{9.80}^{12.33}$	$12.20_{9.80}^{11.83}$	$8.79_{8.43}^{9.47}$	$8.62_{8.32}^{9.09}$
	1000	$0.41_{0.27}^{0.58}$	$0.33_{0.22}^{0.46}$	$10.95_{9.80}^{12.33}$	$10.20_{9.80}^{11.49}$	$8.76_{8.36}^{9.38}$	$8.58_{8.24}^{9.00}$
13 to 18	0	$3.80_{2.67}^{5.05}$	$2.17_{1.16}^{3.36}$	$15.36_{14.56}^{16.61}$	$14.56_{13.57}^{15.36}$	$11.01_{10.58}^{11.60}$	$10.93_{10.53}^{11.42}$
	100	$2.84_{1.92}^{3.80}$	$1.74_{0.96}^{2.64}$	$15.62_{14.70}^{16.61}$	$14.70_{13.57}^{15.62}$	$11.38_{10.88}^{11.93}$	$11.14_{10.67}^{11.68}$
	200	$2.14_{1.36}^{3.07}$	$1.22_{0.71}^{1.82}$	$15.36_{14.56}^{16.61}$	$14.70_{13.86}^{15.75}$	$11.44_{10.88}^{12.02}$	$11.27_{10.74}^{11.87}$
	300	$1.83_{1.10}^{2.71}$	$0.88_{0.51}^{1.36}$	$15.10_{14.14}^{16.25}$	$14.70_{13.57}^{15.75}$	$11.27_{10.74}^{11.87}$	$11.23_{10.67}^{11.83}$
	400	$1.69_{0.99}^{2.54}$	$0.72_{0.41}^{1.15}$	$14.97_{14.00}^{16.13}$	$14.56_{13.57}^{15.75}$	$11.12_{10.59}^{11.70}$	$11.10_{10.56}^{11.70}$
	500	$1.62_{0.94}^{2.45}$	$0.62_{0.37}^{1.02}$	$14.70_{13.57}^{15.75}$	$14.42_{13.27}^{15.36}$	$10.95_{10.43}^{11.52}$	$10.95_{10.41}^{11.54}$
	600	$1.61_{0.91}^{2.43}$	$0.57_{0.34}^{0.95}$	$14.70_{13.57}^{15.75}$	$14.14_{13.27}^{15.10}$	$10.79_{10.29}^{11.36}$	$10.79_{10.21}^{11.38}$
	700	$1.61_{0.91}^{2.42}$	$0.54_{0.33}^{0.93}$	$14.56_{13.42}^{15.62}$	$14.00_{12.81}^{14.97}$	$10.65_{10.16}^{11.21}$	$11.63_{10.05}^{11.23}$
	800	$1.64_{0.94}^{2.45}$	$0.53_{0.32}^{0.91}$	$14.28_{13.42}^{15.36}$	$13.86_{12.81}^{14.97}$	$10.53_{10.05}^{11.06}$	$10.48_{9.92}^{11.06}$
	900	$1.67_{0.98}^{2.50}$	$0.53_{0.32}^{0.92}$	$14.14_{13.27}^{15.36}$	$13.57_{12.81}^{14.70}$	$10.41_{9.92}^{10.95}$	$11.36_{9.75}^{10.93}$
	1000	$1.72_{1.02}^{2.54}$	$0.53_{0.31}^{0.93}$	$14.14_{13.27}^{15.10}$	$13.57_{12.65}^{14.70}$	$10.31_{9.84}^{10.84}$	$10.23_{9.64}^{10.79}$
18 to 23	0	$8.16_{6.83}^{9.59}$	$6.29_{5.01}^{7.66}$	$16.25_{15.62}^{17.21}$	$15.36_{14.70}^{16.25}$	$11.33_{9.84}^{11.74}$	$11.31_{10.97}^{11.75}$
	100	$6.53_{5.43}^{7.70}$	$4.93_{3.91}^{5.98}$	$16.25_{15.62}^{17.21}$	$15.75_{15.23}^{16.61}$	$11.64_{11.33}^{12.04}$	$11.77_{11.40}^{12.25}$
	200	$5.66_{4.56}^{6.82}$	$3.71_{2.83}^{4.78}$	$16.61_{15.75}^{17.55}$	$17.21_{16.25}^{18.22}$	$11.94_{11.54}^{12.45}$	$12.39_{11.89}^{13.02}$
	300	$5.27_{4.16}^{6.43}$	$3.20_{2.29}^{4.28}$	$16.61_{15.75}^{17.55}$	$17.32_{16.25}^{18.55}$	$11.91_{11.46}^{12.46}$	$12.41_{11.87}^{13.04}$
	400	$5.09_{3.99}^{6.24}$	$3.00_{2.05}^{4.07}$	$16.61_{15.75}^{17.55}$	$17.21_{16.25}^{18.33}$	$11.77_{11.31}^{12.34}$	$12.24_{11.70}^{12.88}$
	500	$4.97_{3.90}^{6.13}$	$2.88_{1.92}^{3.97}$	$16.25_{15.36}^{17.21}$	$16.97_{15.75}^{18.11}$	$11.62_{11.17}^{12.20}$	$12.04_{11.50}^{12.67}$
	600	$4.91_{3.85}^{6.06}$	$2.80_{1.84}^{3.91}$	$16.25_{15.36}^{17.21}$	$16.61_{15.62}^{17.66}$	$11.46_{10.99}^{12.02}$	$11.79_{11.27}^{12.43}$
	700	$4.84_{3.82}^{5.97}$	$2.77_{1.82}^{3.90}$	$15.75_{15.10}^{16.97}$	$16.49_{15.36}^{17.55}$	$11.29_{10.84}^{11.87}$	$11.60_{11.10}^{12.24}$
	800	$4.80_{3.82}^{5.89}$	$2.76_{1.82}^{3.88}$	$15.75_{14.97}^{16.73}$	$16.25_{15.10}^{17.32}$	$11.14_{10.70}^{11.72}$	$11.42_{10.93}^{12.04}$
	900	$4.76_{3.82}^{5.79}$	$2.76_{1.83}^{3.88}$	$15.75_{14.70}^{16.73}$	$16.13_{14.97}^{17.21}$	$11.01_{10.58}^{11.56}$	$11.27_{10.77}^{11.87}$
	1000	$4.71_{3.81}^{5.63}$	$2.77_{1.85}^{3.86}$	$15.62_{14.70}^{16.61}$	$15.75_{14.70}^{16.97}$	$10.88_{10.46}^{11.44}$	$11.10_{10.63}^{11.70}$

Supplementary Table S2 Medians, 25th (subscript) and 75th (superscript) percentiles of localization error, FWHM, FVHM (mm) of all FD modes to CW mode at different depth intervals with noise model.

Depth (mm)	Frequency(MHz)	Localization error		FWHM		FVHM	
		NN3	NN4	NN3	NN4	NN3	NN4
3 to 8	0	$0.57_{0.34}^{0.82}$	$0.43_{0.25}^{0.70}$	$9.80_{9.80}^{10.77}$	$9.80_{9.17}^{10.20}$	$8.28_{7.83}^{8.65}$	$8.08_{7.65}^{8.51}$
	100	$0.49_{0.30}^{0.72}$	$0.43_{0.26}^{0.68}$	$9.80_{9.17}^{10.20}$	$9.80_{9.17}^{9.80}$	$8.20_{7.74}^{8.58}$	$8.04_{7.61}^{8.47}$
	200	$0.43_{0.26}^{0.66}$	$0.42_{0.26}^{0.65}$	$9.80_{9.17}^{10.00}$	$9.80_{9.17}^{9.80}$	$8.04_{7.56}^{8.47}$	$7.96_{7.51}^{8.43}$
	300	$0.43_{0.28}^{0.62}$	$0.42_{0.27}^{0.62}$	$9.80_{9.17}^{9.80}$	$9.38_{9.17}^{9.80}$	$7.92_{7.42}^{8.36}$	$7.83_{7.37}^{8.32}$
	400	$0.45_{0.30}^{0.62}$	$0.44_{0.30}^{0.62}$	$9.17_{8.94}^{9.80}$	$9.17_{8.94}^{9.80}$	$7.79_{7.27}^{8.28}$	$7.74_{7.27}^{8.20}$
	500	$0.48_{0.34}^{0.64}$	$0.47_{0.33}^{0.63}$	$9.17_{8.94}^{9.80}$	$9.17_{8.94}^{9.80}$	$7.61_{7.17}^{8.16}$	$7.61_{7.11}^{8.08}$
	600	$0.51_{0.36}^{0.67}$	$0.50_{0.36}^{0.66}$	$9.17_{8.94}^{9.80}$	$9.17_{8.94}^{9.80}$	$7.51_{7.01}^{8.04}$	$7.47_{7.01}^{7.96}$
	700	$0.54_{0.39}^{0.70}$	$0.53_{0.38}^{0.69}$	$9.17_{8.72}^{9.80}$	$9.17_{8.49}^{9.80}$	$7.42_{6.95}^{7.92}$	$7.37_{6.90}^{7.87}$
	800	$0.58_{0.42}^{0.75}$	$0.56_{0.40}^{0.73}$	$9.17_{8.49}^{9.80}$	$8.17_{8.49}^{9.38}$	$7.32_{6.90}^{7.83}$	$7.27_{6.84}^{7.74}$
	900	$0.61_{0.44}^{0.80}$	$0.59_{0.42}^{0.78}$	$9.17_{8.49}^{9.80}$	$9.17_{8.49}^{9.38}$	$7.27_{6.84}^{7.79}$	$7.17_{6.78}^{7.65}$
	1000	$0.65_{0.46}^{0.85}$	$0.64_{0.45}^{0.84}$	$9.17_{8.49}^{9.80}$	$9.17_{8.49}^{9.38}$	$7.22_{6.78}^{7.70}$	$7.11_{6.72}^{7.56}$
8 to 13	0	$1.04_{0.60}^{1.69}$	$0.64_{0.36}^{0.95}$	$13.27_{12.00}^{14.70}$	$12.65_{11.49}^{13.57}$	$10.00_{9.38}^{10.63}$	$9.84_{9.16}^{10.46}$
	100	$0.76_{0.44}^{1.21}$	$0.62_{0.35}^{0.91}$	$13.12_{12.00}^{14.56}$	$12.33_{11.49}^{13.57}$	$10.00_{9.28}^{10.65}$	$9.73_{9.06}^{10.36}$
	200	$0.57_{0.32}^{0.91}$	$0.55_{0.31}^{0.83}$	$12.81_{11.49}^{14.14}$	$12.33_{11.31}^{13.42}$	$9.81_{9.06}^{10.53}$	$9.55_{8.96}^{10.23}$
	300	$0.52_{0.30}^{0.83}$	$0.52_{0.30}^{0.80}$	$12.33_{11.31}^{13.86}$	$12.00_{10.77}^{13.27}$	$9.58_{8.93}^{10.34}$	$9.38_{8.83}^{10.05}$
	400	$0.52_{0.31}^{0.83}$	$0.51_{0.30}^{0.81}$	$12.33_{10.77}^{13.57}$	$12.00_{10.77}^{13.27}$	$9.41_{8.79}^{10.13}$	$9.25_{8.72}^{9.89}$
	500	$0.55_{0.34}^{0.87}$	$0.54_{0.33}^{0.84}$	$12.00_{10.77}^{13.42}$	$11.83_{10.39}^{12.96}$	$9.28_{8.69}^{9.97}$	$9.13_{8.62}^{9.75}$
	600	$0.59_{0.37}^{0.93}$	$0.58_{0.37}^{0.91}$	$12.00_{10.77}^{13.42}$	$11.66_{10.20}^{12.96}$	$9.16_{8.62}^{9.86}$	$9.00_{8.51}^{9.64}$
	700	$0.66_{0.42}^{1.04}$	$0.64_{0.42}^{0.99}$	$12.00_{10.39}^{13.42}$	$11.66_{10.20}^{12.96}$	$9.06_{8.51}^{9.75}$	$8.90_{8.43}^{9.52}$
	800	$0.74_{0.49}^{1.20}$	$0.73_{0.48}^{1.13}$	$11.83_{10.39}^{13.42}$	$11.49_{10.20}^{13.12}$	$8.93_{8.40}^{9.61}$	$8.79_{8.28}^{9.44}$
	900	$0.86_{0.57}^{1.45}$	$0.84_{0.56}^{1.32}$	$11.83_{10.39}^{13.57}$	$11.49_{10.20}^{13.27}$	$8.83_{8.28}^{9.52}$	$8.69_{8.16}^{9.31}$
	1000	$1.02_{0.66}^{1.89}$	$0.98_{0.65}^{1.63}$	$11.83_{10.39}^{13.57}$	$11.49_{10.20}^{13.27}$	$8.69_{8.16}^{9.41}$	$8.55_{8.04}^{9.22}$
13 to 18	0	$4.21_{2.90}^{5.83}$	$2.39_{1.38}^{3.64}$	$16.25_{14.70}^{18.22}$	$14.97_{14.00}^{16.61}$	$10.99_{10.48}^{11.70}$	$10.90_{10.43}^{11.50}$
	100	$3.22_{2.17}^{4.52}$	$2.03_{1.22}^{3.03}$	$16.37_{14.97}^{18.33}$	$15.36_{14.00}^{17.21}$	$11.27_{10.70}^{11.96}$	$11.06_{10.56}^{11.70}$
	200	$2.47_{1.57}^{3.59}$	$1.55_{0.97}^{2.34}$	$16.25_{14.70}^{18.11}$	$15.36_{14.14}^{17.44}$	$11.31_{10.72}^{12.04}$	$11.19_{10.63}^{11.91}$
	300	$2.14_{1.31}^{3.21}$	$1.29_{0.81}^{1.99}$	$15.75_{14.56}^{17.66}$	$15.36_{14.00}^{17.55}$	$11.17_{10.56}^{11.89}$	$11.14_{10.56}^{11.87}$
	400	$2.04_{1.22}^{3.11}$	$1.21_{0.77}^{1.89}$	$15.75_{14.14}^{17.66}$	$15.36_{14.00}^{17.55}$	$10.99_{10.39}^{11.72}$	$11.01_{10.41}^{11.75}$
	500	$2.06_{1.22}^{3.20}$	$1.21_{0.77}^{1.91}$	$15.62_{14.00}^{17.66}$	$15.36_{13.57}^{17.55}$	$10.81_{10.18}^{11.54}$	$10.88_{10.23}^{11.62}$
	600	$2.14_{1.26}^{3.46}$	$1.29_{0.82}^{2.03}$	$15.62_{14.00}^{18.00}$	$15.36_{13.57}^{17.55}$	$10.63_{10.00}^{11.38}$	$10.67_{10.05}^{11.46}$
	700	$2.36_{1.37}^{4.21}$	$1.42_{0.89}^{2.27}$	$15.62_{13.57}^{18.11}$	$15.36_{13.57}^{18.00}$	$10.43_{9.78}^{11.21}$	$10.53_{9.86}^{11.31}$
	800	$2.84_{1.57}^{41.22}$	$1.64_{1.01}^{2.75}$	$15.36_{13.57}^{18.33}$	$15.36_{13.57}^{18.22}$	$10.23_{9.58}^{11.04}$	$10.36_{9.70}^{11.17}$
	900	$5.28_{1.92}^{75.42}$	$2.04_{1.21}^{4.65}$	$15.36_{13.42}^{18.55}$	$15.36_{13.42}^{18.55}$	$10.05_{9.38}^{10.88}$	$10.18_{9.52}^{11.01}$
	1000	$49.54_{2.88}^{89.40}$	$3.07_{1.54}^{60.00}$	$15.36_{13.27}^{18.55}$	$15.36_{13.42}^{18.87}$	$9.89_{9.19}^{10.74}$	$10.03_{9.31}^{10.86}$
18 to 23	0	$54.99_{9.12}^{92.23}$	$7.38_{5.46}^{30.18}$	$18.22_{16.49}^{20.59}$	$16.61_{15.36}^{18.76}$	$11.48_{10.97}^{12.18}$	$11.40_{10.90}^{12.06}$
	100	$20.35_{7.00}^{82.97}$	$6.35_{4.57}^{10.80}$	$18.33_{16.61}^{20.88}$	$17.55_{15.75}^{20.10}$	$11.77_{11.14}^{12.50}$	$11.81_{11.21}^{12.58}$
	200	$8.44_{5.56}^{68.85}$	$4.96_{3.44}^{7.69}$	$18.55_{16.61}^{21.26}$	$18.87_{16.61}^{22.00}$	$12.02_{11.36}^{12.82}$	$12.29_{11.56}^{13.17}$
	300	$7.86_{5.07}^{68.82}$	$4.34_{2.89}^{6.86}$	$18.55_{16.61}^{21.35}$	$19.08_{16.73}^{22.36}$	$11.96_{11.25}^{12.83}$	$12.29_{11.56}^{13.18}$
	400	$8.98_{5.02}^{77.94}$	$4.22_{2.72}^{7.04}$	$18.55_{16.49}^{21.35}$	$19.08_{16.73}^{22.36}$	$11.77_{11.06}^{12.62}$	$12.13_{11.40}^{13.01}$
	500	$37.05_{5.37}^{87.49}$	$4.34_{2.70}^{9.47}$	$18.55_{16.25}^{21.63}$	$19.08_{16.73}^{22.45}$	$11.54_{10.81}^{12.41}$	$11.91_{11.19}^{12.78}$
	600	$56.29_{6.60}^{92.40}$	$4.88_{2.83}^{53.68}$	$18.55_{16.25}^{21.91}$	$18.97_{16.61}^{22.45}$	$11.33_{10.58}^{12.18}$	$11.66_{10.93}^{12.53}$
	700	$66.14_{25.60}^{95.81}$	$7.60_{3.19}^{73.68}$	$18.55_{15.75}^{22.18}$	$18.97_{16.37}^{22.72}$	$11.08_{10.29}^{11.98}$	$11.44_{10.70}^{12.32}$
	800	$71.30_{40.08}^{97.27}$	$44.79_{4.12}^{84.23}$	$19.08_{16.13}^{23.07}$	$19.08_{16.25}^{22.98}$	$10.97_{10.13}^{11.98}$	$11.25_{10.48}^{12.15}$
	900	$73.86_{45.95}^{98.31}$	$60.20_{10.40}^{90.53}$	$19.39_{16.25}^{23.64}$	$19.29_{16.25}^{23.15}$	$10.99_{10.13}^{11.94}$	$11.12_{10.34}^{12.00}$
	1000	$75.05_{48.27}^{98.81}$	$67.55_{34.90}^{93.87}$	$20.10_{16.61}^{24.90}$	$19.29_{16.25}^{23.58}$	$11.12_{10.21}^{12.18}$	$11.01_{10.21}^{11.91}$

Supplementary Table S3 Improvement of localization error, FWHM, FVHM (%) of all FD modes to CW mode at different depth intervals without noise model. ($p<0.0001$)

Depth (mm)	Frequency (MHz)	Localization error		FWHM		FVHM	
		NN3	NN4	NN3	NN4	NN3	NN4
3 to 8	100	17.65	2.24	0.00	0.00	0.95	0.51
	200	29.11	7.91	0.00	4.26	2.90	2.06
	300	31.48	10.99	4.26	6.46	4.93	3.67
	400	30.80	11.62	6.46	6.46	6.52	4.77
	500	27.51	8.88	6.46	6.46	8.16	5.90
	600	25.38	4.14	6.46	8.71	9.29	7.64
	700	21.55	0.19	8.71	8.71	10.44	8.84
	800	18.13	-2.96	8.71	8.71	11.63	10.70
	900	16.21	-5.51	8.71	8.71	12.85	11.99
	1000	14.25	-7.20	8.71	8.71	14.10	13.31
8 to 13	100	32.85	10.12	2.36	1.32	0.00	1.13
	200	55.77	25.90	3.55	1.32	2.14	2.88
	300	61.51	35.29	7.24	2.67	4.68	5.01
	400	63.01	40.74	8.50	5.41	7.05	6.91
	500	63.01	42.71	8.50	6.81	8.59	8.55
	600	62.26	42.10	12.40	8.23	9.86	9.91
	700	60.91	40.88	12.40	12.64	11.18	10.96
	800	59.59	39.99	13.73	17.28	11.85	11.67
	900	57.30	36.56	13.73	17.28	12.53	12.40
	1000	55.45	34.37	16.48	17.28	12.87	12.76
13 to 18	100	25.47	19.70	-1.68	-0.94	-3.28	-2.00
	200	43.79	43.72	0.00	-0.94	-3.84	-3.17
	300	51.92	59.39	1.71	-0.94	-2.33	-2.78
	400	55.45	66.92	2.57	0.00	-0.99	-1.61
	500	57.31	71.37	4.33	0.95	0.61	-0.20
	600	57.81	73.88	4.33	2.87	2.04	1.24
	700	57.82	74.95	5.22	3.85	3.30	2.73
	800	56.98	75.49	7.02	4.84	4.39	4.05
	900	55.85	75.61	7.94	6.83	5.48	5.17
	1000	54.76	75.74	7.94	6.83	6.39	6.32
18 to 25	100	20.06	21.63	0.00	-2.51	-2.67	-4.07
	200	30.72	41.06	-2.25	-12.00	-5.38	-9.56
	300	35.47	49.12	-2.25	-12.75	-5.06	-9.71
	400	37.66	52.36	-2.25	-12.00	-3.88	-8.16
	500	39.09	54.22	0.00	-10.47	-2.51	-6.40
	600	39.87	55.48	0.00	-8.14	-1.09	-4.24
	700	40.69	55.91	3.08	-7.36	0.37	-2.52
	800	41.16	56.10	3.08	-5.77	1.68	-0.92
	900	41.71	56.13	3.08	-4.97	2.82	0.37
	1000	42.34	56.02	3.87	-2.51	4.01	1.87

Supplementary Table S4 Improvement of localization error, FWHM, FVHM (%) of all FD modes to CW mode at different depth intervals with noise model. ($p<0.0001$)

Depth (mm)	Frequency (MHz)	Localization error		FWHM		FVHM	
		NN3	NN4	NN3	NN4	NN3	NN4
3 to 8	100	14.12	0.04	0.00	0.00	0.95	0.51
	200	24.53	3.32	0.00	0.00	2.90	1.54
	300	24.95	2.24	0.00	4.26	4.42	3.13
	400	21.39	-1.88	6.46	6.46	5.98	4.22
	500	15.63	-8.31	6.46	6.46	8.16	5.90
	600	10.23	-15.76	6.46	6.46	9.29	7.64
	700	4.21	-22.90	6.46	6.46	10.44	8.84
	800	-2.14	-30.15	6.46	6.46	11.63	10.07
	900	-7.90	-37.57	6.46	6.46	12.23	11.34
	1000	-14.87	-47.27	6.46	6.46	12.85	11.99
8 to 13	100	26.99	3.90	1.14	2.53	0.00	1.13
	200	45.54	13.96	3.47	2.53	1.90	2.88
	300	50.02	18.89	7.06	5.13	4.17	4.70
	400	50.10	19.76	7.06	5.13	5.95	5.95
	500	47.39	16.46	9.54	6.46	7.17	7.23
	600	43.38	9.10	9.54	7.80	8.42	8.55
	700	36.58	-0.27	9.54	7.80	9.39	9.57
	800	28.55	-13.77	10.81	9.17	10.71	10.61
	900	17.03	-30.22	10.81	9.17	11.72	11.67
	1000	1.88	-52.32	10.81	9.17	13.11	13.13
13 to 18	100	23.47	14.84	-0.76	-2.64	-2.54	-1.42
	200	41.28	34.98	0.00	-2.64	-2.92	-2.60
	300	49.02	45.95	3.08	-2.64	-1.58	-2.21
	400	51.41	49.45	3.08	-2.64	0.00	-1.02
	500	51.11	49.18	3.87	-2.64	1.64	0.21
	600	49.09	45.95	3.87	-2.64	3.32	2.10
	700	43.99	40.69	3.87	-2.64	5.08	3.41
	800	32.55	31.33	5.45	-2.64	6.90	4.98
	900	-25.50	14.77	5.45	-2.64	8.54	6.60
	1000	-1077.71	-28.44	5.45	-2.64	10.01	8.03
18 to 25	100	62.98	13.97	-0.60	-5.64	-2.58	-3.65
	200	84.65	32.70	-1.79	-13.57	-4.70	-7.84
	300	85.71	41.16	-1.79	-14.84	-4.23	-7.84
	400	83.67	42.78	-1.79	-14.84	-2.58	-6.42
	500	32.62	41.09	-1.79	-14.84	-0.52	-4.48
	600	-2.38	33.90	-1.79	-14.21	1.25	-2.29
	700	-20.28	-2.98	-1.79	-14.21	3.48	-0.36
	800	-29.66	-507.29	-4.71	-14.84	4.43	1.28
	900	-34.33	-716.29	-6.42	-16.10	4.23	2.40
	1000	-36.49	-815.93	-10.31	-16.10	3.09	3.35

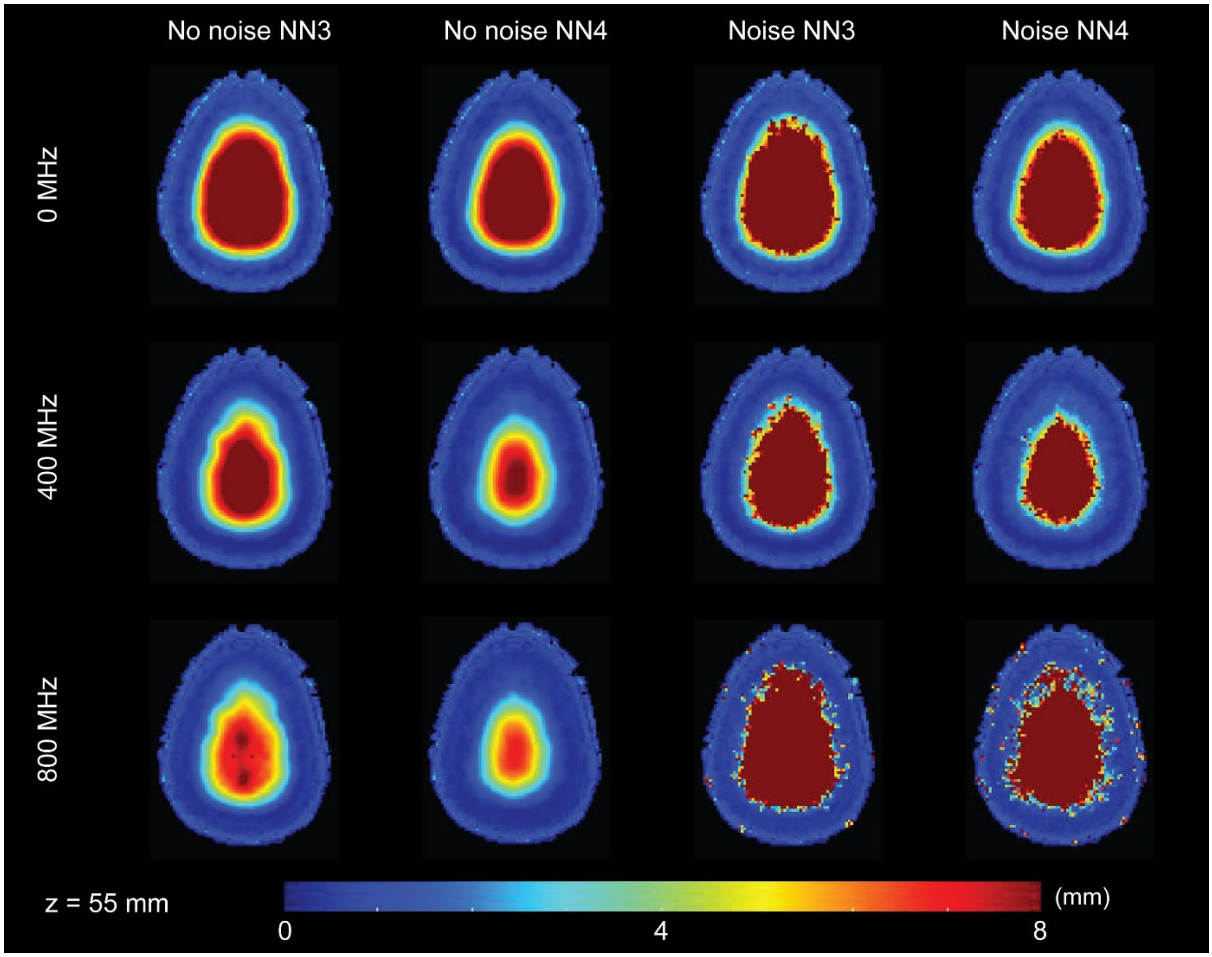

Supplementary Figure S1 | Localization error distribution in an example transverse slice of HbR. The color of each voxel reflects the localization error in mm averaged across five head models after spatial alignment. For brevity, three modulation frequencies are shown: 0, 400, 800 MHz.

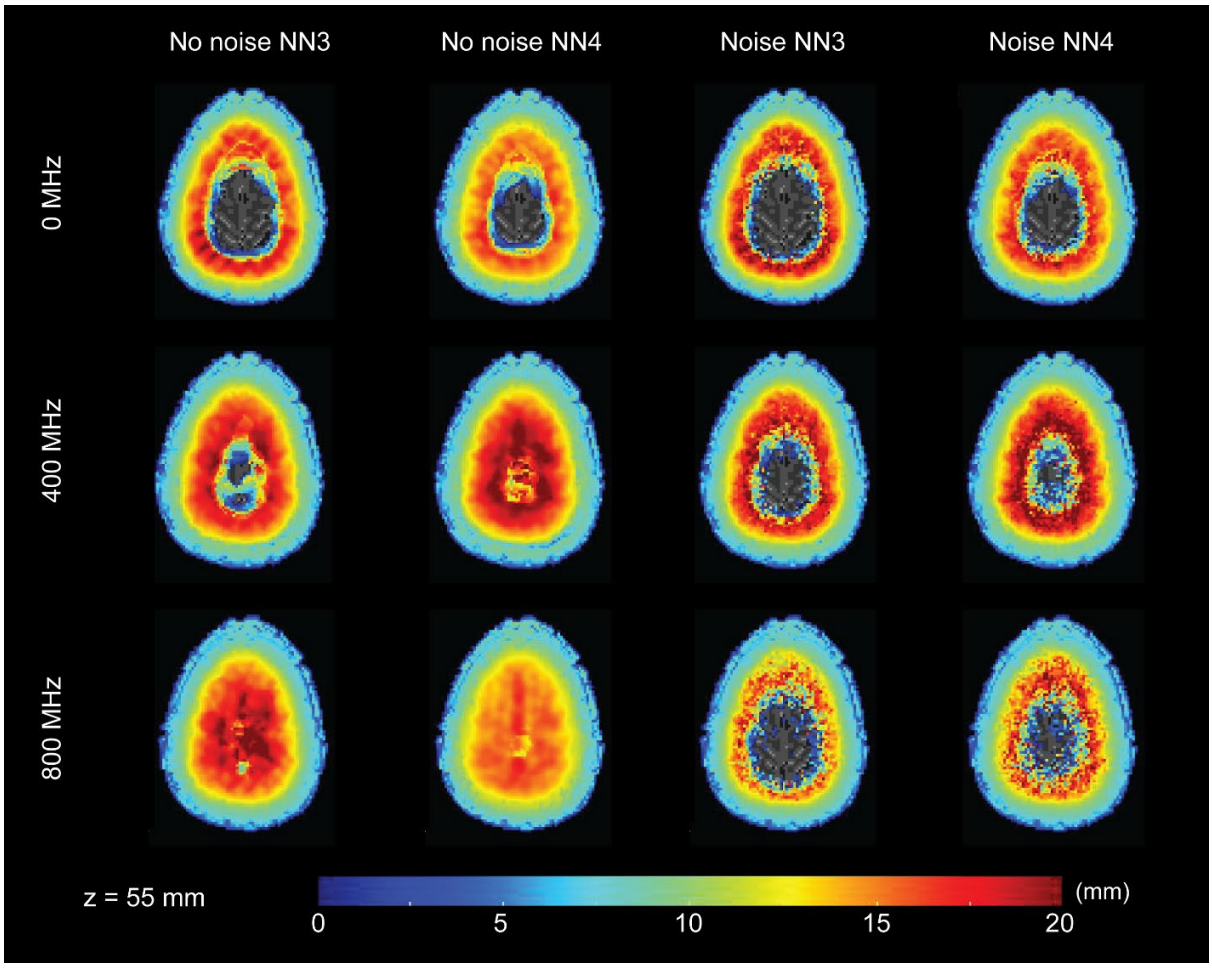

Supplementary Figure S2 | FWHM distribution in an example transverse slice of HbR. The color of each voxel reflects the FWHM in mm averaged across five head models after spatial alignment. For brevity, three modulation frequencies are shown: 0, 400, 800 MHz.

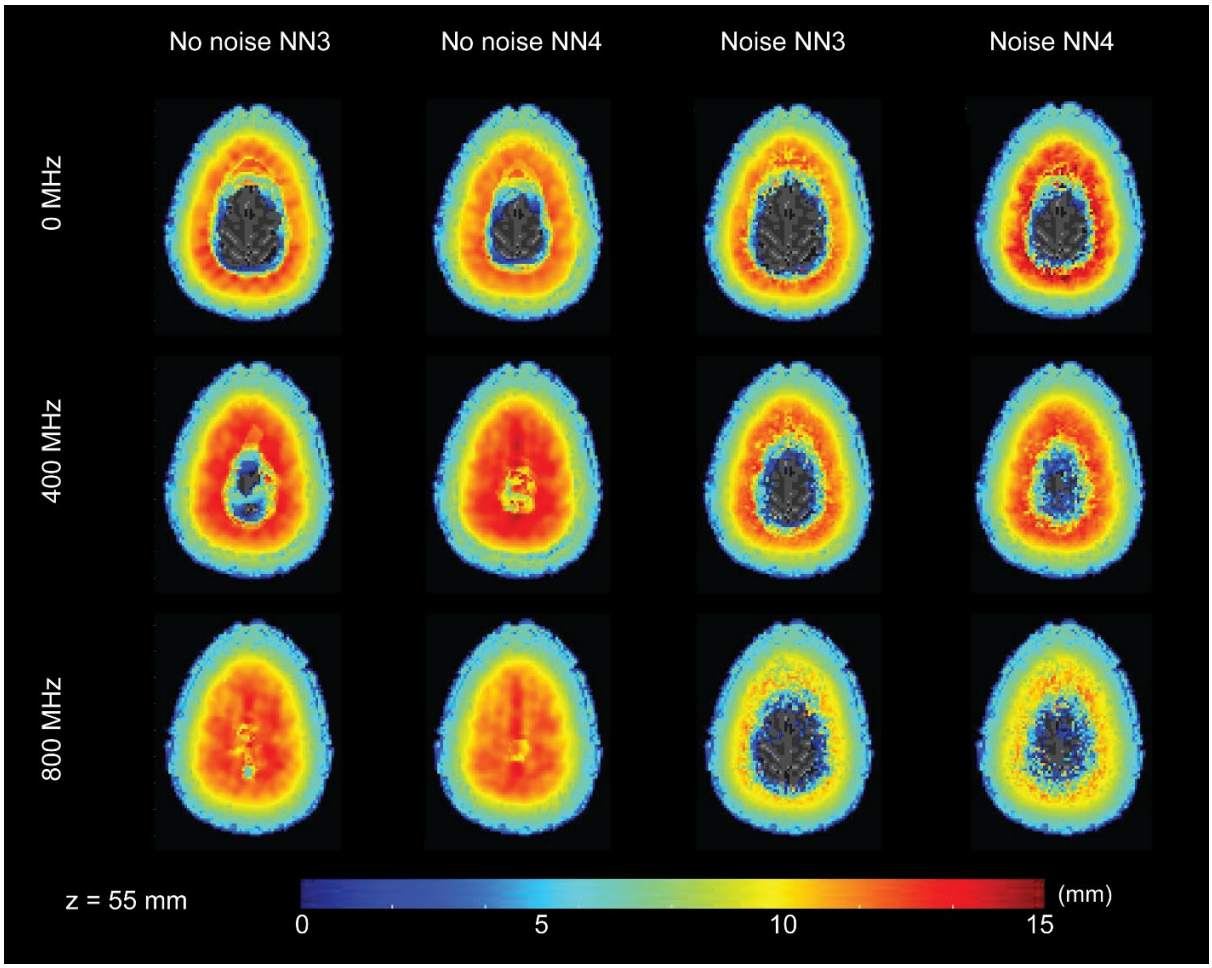

Supplementary Figure S3 | Spatial distribution of the cube root of the FVHM in an example transverse slice of HbR. The color of each voxel reflects the cube root of the FVHM in mm averaged across five head models after spatial alignment. For brevity, three modulation frequencies are shown: 0, 400, 800 MHz.

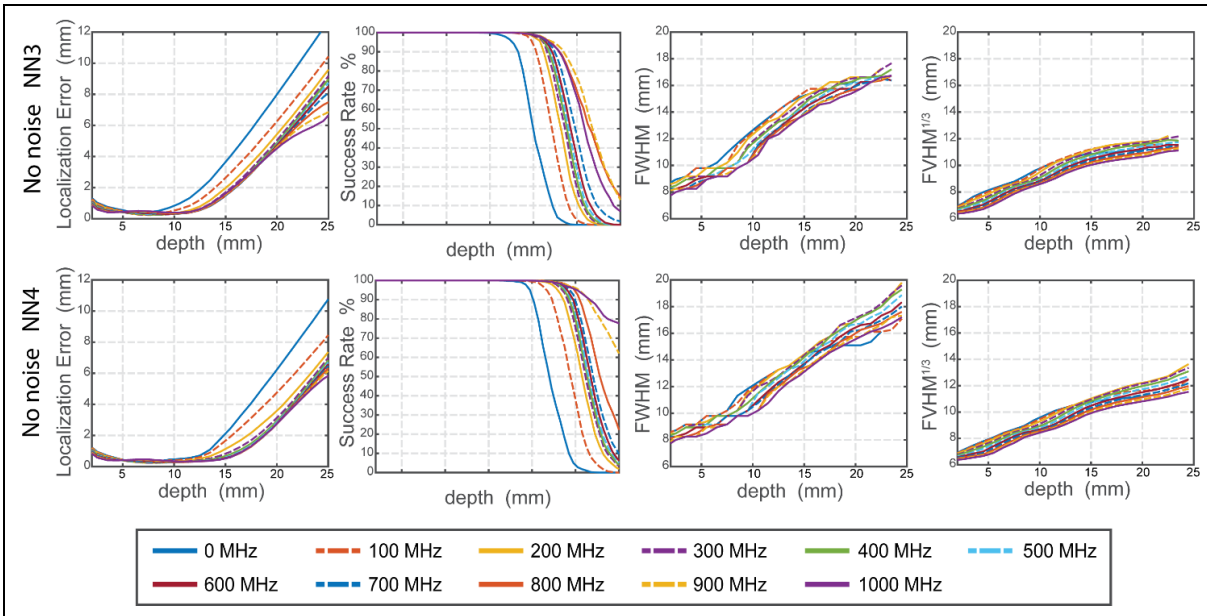

Supplementary Figure S4 | Image quality metrics as a function of depth below the surface in noise free cases of HbR. The median of the localization error, success rate FWHM, cube root of FVHM across 5 head models' simulated measurements of 11 modulation frequencies without noise added for NN3 and NN4. Even frequencies are solid lines and odd frequencies are dashed lines.

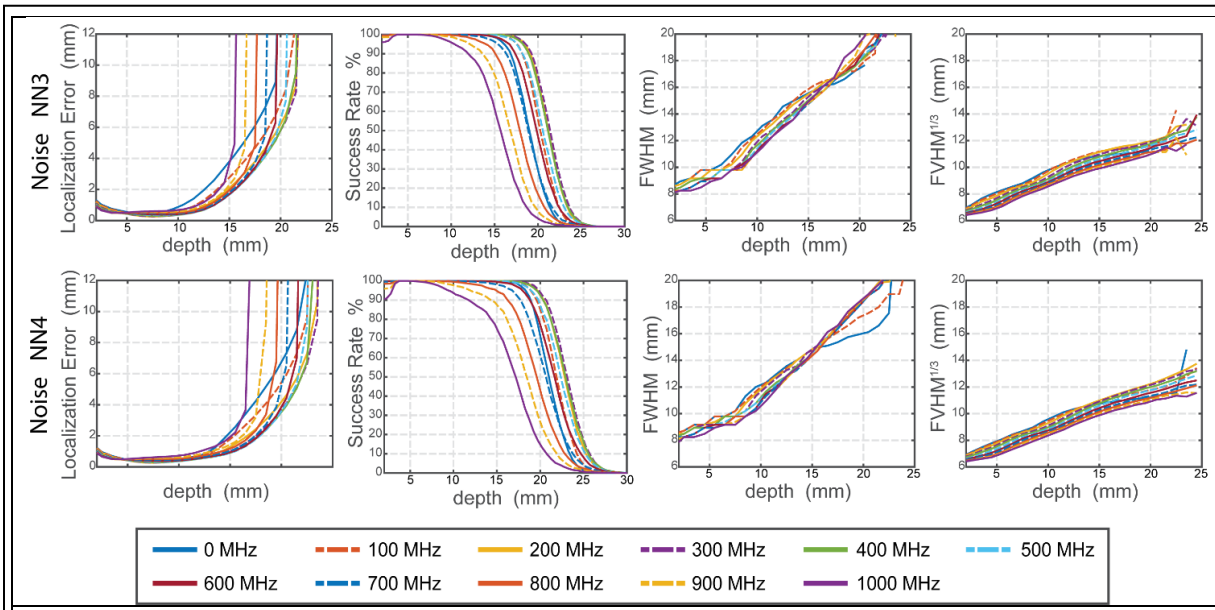

Supplementary Figure S5 | Image quality metrics as a function of depth below the surface in noise added cases of HbR. The median of the localization error, success rate FWHM, cube root of FVHM across 5 head models' simulated measurements of 11 modulation frequencies without noise added for NN3 and NN4. Even frequencies are solid lines and odd frequencies are dashed lines.

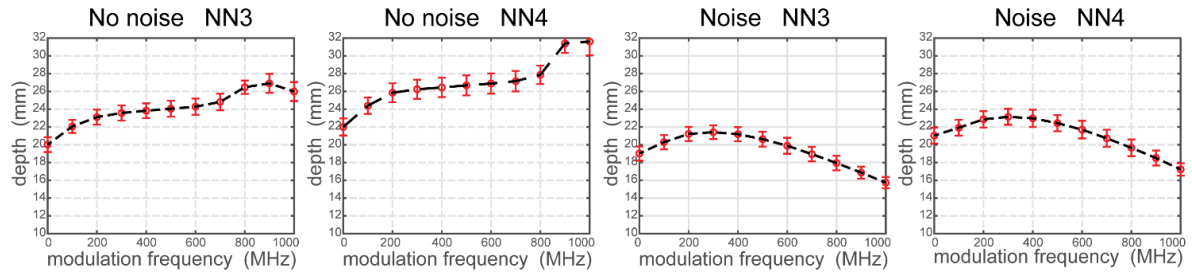

Supplementary Figure S6 | Depth of the reliable field of view across all modulation frequencies and noise models of HbR. Averaged depths of the 50% success rate as a function of modulation frequency. Error bars represent standard deviations across five head models.
